# Comparative Transcriptome Analysis of Male Sterile Anthers Induced by High Temperature in Wheat (*Triticum aestivum* L.)

**DOI:** 10.3389/fpls.2021.727966

**Published:** 2021-10-25

**Authors:** Hongzhan Liu, Zhongke Sun, Lizong Hu, Chaoqiong Li, Xueqin Wang, Zonghao Yue, Yulin Han, Guangyu Yang, Keshi Ma, Guihong Yin

**Affiliations:** ^1^College of Life Science and Agronomy, Zhoukou Normal University, Zhoukou, China; ^2^College of Agronomy, Henan Agricultural University, Zhengzhou, China; ^3^Zhoukou Academy of Agricultural Sciences, Zhoukou, China

**Keywords:** wheat, anther transcriptome, high temperature, male sterility, hub gene

## Abstract

Global warming will have a negative effect on agricultural production as high temperature (HT) stress can seriously threaten plant growth and reproduction. Male sterility caused by HT may be exploited by the creation of a male-sterile line, which has great potential for application in crop heterosis. Therefore, it is important to understand the molecular mechanisms of anther abortion induced by HT in wheat, which remain unclear at present. In this study, we performed phenotype improve language in the abstract and comparative transcriptome analysis of the male sterile anthers induced by HT in wheat. Compared with Normal anthers, the cytological analysis indicated that HT-induced male sterile anthers were smaller and had no starch accumulation in pollen grains, which is consistent with the results observed by scanning electron microscopy (SEM). The 9601 differentially expressed genes (DEGs) identified by transcriptome sequencing compared with the Normal anthers were noticeably involved in the following pathways: starch and sucrose metabolism, phosphatidylinositol (PI) signaling system, peroxidase activity and response to oxidative stress, and heme binding. In addition, TUNEL assays were performed and the results further confirmed the excessive accumulation of reactive oxygen species (ROS) in sterile anthers. Moreover, a total of 38 hub genes were obtained from the protein-protein interaction network analysis of these pathways, including genes, for example, heat shock protein 90 (HSP90), thioredoxin-like protein 1, peroxidase (POD), calreticulin, UDP glucose pyrophosphorylase (UGPase), sucrose synthase, phosphatidylinositol-4-phosphate 5-Kinase (PIP5K), cytochrome c, and Cystathionine beta-synthase X6-like (CBSX6-like). These findings provide insights for predicting the functions of the candidate genes, and the comprehensive analysis of our results is helpful for studying the abortive interaction mechanism induced by HT in wheat.

## Introduction

Heterosis is widely used to increase crop yield, where the offspring derived from crosses between different inbred lines have superior yield performance than their respective parents. Yields of major crops, such as rice, maize, sorghum, and other species, have significantly improved through crossbreeding, up to 15–50%, compared with inbred varieties (Edgerton, 2009; Sun et al., 2018). Thus, heterosis has brought tremendous economic benefits to global crop production (Liu et al., 2018c). As one of the top three global crops, wheat is a critical cereal and the most widely grown crop in the world, supplying nearly 20% of the daily food of the world (Brenchley et al., 2012). However, the production of sufficient amounts of hybrid seeds in self-pollinating wheat is more challenging. Until now, the most effective way to increase wheat yield is still through short-term hybrid breeding. Therefore, the mechanism of male sterility, which is the basis of heterosis utilization in wheat, has long been a focus of wheat research.

Global warming will have a negative impact on agricultural production as high temperature (HT) stress can seriously threaten plant growth and reproduction (Peng et al., 2004). The anther development of a plant is more sensitive to abiotic stress than vegetative growth, such as heat stress, cold stress, and drought stress (Storme and Geelen, 2014). For decades, male sterility caused by HT has been known to reduce yield. On the other hand, temperature-sensitive genic male sterility could be applied to production practice as a powerful tool for heterosis, but little is known about the mechanism of HT-induced male sterility.

Male-sterile lines can be created by HT induction, which may be of potential for application in crop heterosis (Liu et al., 2018a). In fact, HT-induced male sterility had been reported in various plants. A study of the barley (*Hordeum vulgare*) has suggested that HTs of 30°C (day) and 25°C (night) can cause infertility (Abiko et al., 2005). In rice, the shape of most pollen grains in HT-treated plants is normal, but the germination on the stigma of these pollen grains is poor (Endo et al., 2009). In heat-sensitive tomato cultivars, the number of pollen grains is reduced, their viability and germinability are impaired, and the fruit set and the number of seeds per fruit are markedly reduced by higher temperature (Firon et al., 2006). Recent studies have found that HT-induced male sterility in HT-sensitive cotton line is characterized by an indehiscent anther wall with abortive pollen grains, but the HT-tolerant cotton line shows normally developed anthers and pollen grains at the same ambient temperature (Ma et al., 2018). Moreover, in the model plant *Arabidopsis thaliana*, the development of pollen within the anther is affected by heat shock at 42°C (Sang et al., 2001). A recent study has shown that the male sterility caused by exposure to higher temperatures could be reduced by the auxin application (Abbas et al., 2018).

In wheat, several studies have shown that when wheat spikelets are exposed to HT stress between ear initiation and anthesis, it causes abnormal pollen grains and male sterility in the anthers (Saini et al., 1984; Dawson and Wardlaw, 1989; Vu et al., 2018). Furthermore, our previous research also suggests that the physiological male-sterile plants of wheat established using a temperature underneath a plastic film of ~10°C higher than the outside temperature, exhibit complete male sterility (Liu et al., 2018a). Therefore, HT may be a convenient way for the breeder to choose an appropriate hybrid combination, contrasting to the traditional way that needs artificial emasculation. Although HT-induced male sterility has been known for years, the mechanisms by which HT-induced male sterility occurs in wheat are not well understood. Thus, in view of the research outlined above and in order to further study the mechanism of sterility caused by HT, we explored phenotypic changes and performed comparative transcriptome analysis of the male sterile anthers induced by HT in wheat.

## Materials and Methods

### Plant Materials, Growth, Experimental Conditions, and Anther Collection

The wheat cultivar Zhoumai 28 was grown in the Yulanyuan experimental base of Zhoukou Normal University in Zhoukou, Henan Province, People's Republic of China (33° 64′ N, 114° 6′ E) on October 13, 2017. We divided the experimental field into two plots, with a row length of 50 cm, row width of 15 cm, and 50 rows as a plot. For both plots, a piece of transparent plastic film supported by thin steel pipes and plastic joints was set up to shed wheat plants when necessary. At the start of April, the wheat had reached Feekes growth stage 8.5 (Liu et al., 2018a), the inner morphology of the wheat ears had developed to the differentiation of stamen and pistils, and the outer morphology was shown as the flag leaves half as long as the penultimate leaves. Plants from one plot were covered by a transparent plastic film to induce HT and those from the other plot were left uncovered as a control. If there is rain, both plots were covered by the film to avoid potential differences introduced by rain between the two plots. Glass mercury thermometers are placed inside and outside the shed to monitor the temperature every day. We checked and recorded the temperature in the morning (8:00), noon (14:00), and evening (20:00). The average temperature underneath the plastic film was ~10°C higher than the outside temperature (more details are available in Supplementary Figure 1).

### Histological Analyses and Phenotype Characteristics

The bright-field photographs of individual spikelets (including seed), flowers, and anthers were captured with a Canon DS126621 digital camera (Canon, Japan); details were described previously (Liu et al., 2018b). The trinuclear stage anthers from both Normal and HT plants were collected from spikelets just prior to anthesis. In detail, Normal anthers and high temperature-male sterile (HT-ms) anthers were stripped on ice and collected in 2-ml centrifuge tubes when they had developed to the trinuclear stage. The whole spikelets were stripped from the uppermost, lowermost, and middle florets, and only the anthers were taken from the two side florets of the middle segment, which had the same development. Pollen grains were stained with iodine potassium iodide solution (1% I_2_-KI) on microscope slides. Then, the microscope slides were observed and photographed by the microscope (Nikon ECLIPSE E100, Japan). For scanning electron microscopy (SEM), freeze-dried anthers and pollen grains were coated with palladium-gold in a vacuum coating machine and detailed operations according to Zhang et al. (2007), and then observed with an FEI Quanta 200 scanning electron microscope (FEI Company, Eindhoven, The Netherlands) with an acceleration voltage of 15 kV and photographed.

### Roche-Tunel Assay

The trinuclear stage anthers from both Normal and HT plants were collected and fixed with FAA (40% formaldehyde 4 ml, acetic acid 6 ml, 50% ethanol 90 ml) for 48 h, and then embedded in paraffin. The paraffin-embedded wheat anthers were cut into 12 μm slices with the microtome (Leica RM2016, Germany). Then briefly, transverse sections of these anthers were first soaked in xylene for 15 min and two times for wax removal, followed by washes in a series of ethanol concentrations (100%, 100%, 85%, 75%, 50%, 30%, and distilled water), each for 5 min. Once the slices were slightly dried, a circle was drawn around the tissue (to prevent the liquid from running away) with a pap pen, then treated with proteinase K^+^ (Promega) and incubated at 37°C for 25 min, then washed in phosphate buffered saline (PBS) for 5 min and three times. Subsequently, a TUNEL assay was performed using an *in situ* cell death detection kit fluorescein (Roche, Mannheim, Germany). Appropriate reagents TdT and dUTP in the kit were mixed in the ratio of 1:9, incubated the slices for 2 h at 37°C. Then, the slices were washed in PBS and were counterstained in DAPI (4′,6-diamidino-2-phenylindole) for 10 min in the dark, and then rinsed three times with PBS. The stained anther sections were observed with a positive fluorescence microscope (Nikon ECLIPSE C1, Japan) under UV light for TUNEL signal and DAPI staining at 380 nm (excitation) and 420 nm (emission), and at 480 nm (excitation) and 555 nm (emission), respectively. The resulting images were processed and analyzed using imaging software Zeiss AxioVision 4.8 (Zeiss, Germany).

### RNA-Sequencing

#### RNA Quantification and Qualification

The RNA concentration was measured using NanoDrop 2000 (Thermo), and the RNA integrity was assessed using the RNA Nano 6000 Assay Kit of the Agilent Bioanalyzer 2100 system (Agilent Technologies, CA, USA).

#### Library Preparation for Transcriptome Sequencing

A total amount of 1 μg RNA per sample was used as input material for the RNA sample preparations. Sequencing libraries were generated using NEBNext UltraTM RNA Library Prep Kit for Illumina (NEB, USA) following the recommendations of the manufacturer, and index codes were added to attribute sequences to each sample. Briefly, mRNA was purified from total RNA using poly-T oligo-attached magnetic beads. Fragmentation was carried out using divalent cations under elevated temperature in NEBNext First Strand Synthesis Reaction Buffer (5X). First strand cDNA was synthesized using random hexamer primer and M-MuLV Reverse Transcriptase. Second strand cDNA synthesis was subsequently performed using DNA Polymerase I and RNase H. Remaining overhangs were converted into blunt ends *via* exonuclease/polymerase activities. After adenylation of 3′ ends of DNA fragments, NEBNext Adaptor with hairpin loop structure was ligated to prepare for hybridization. In order to select cDNA fragments of preferentially 240 bp in length, the library fragments were purified with the AMPure XP system (Beckman Coulter, Beverly, USA). Then 3 μl USER Enzyme (NEB, USA) was used with size-selected, adaptor-ligated cDNA at 37°C for 15 min followed by 5 min at 95°C before PCR. Then, PCR was performed with Phusion High-Fidelity DNA polymerase, Universal PCR primers, and Index (X) Primer. At last, PCR products were purified (AMPure XP system) and library quality was assessed on the Agilent Bioanalyzer 2100 system.

#### Clustering and Sequencing

The clustering of the index-coded samples was performed on a cBot Cluster Generation System using TruSeq PE Cluster Kit v4-cBot-HS (Illumia) according to the instructions of the manufacturer. After cluster generation, the library preparations were sequenced on an Illumina platform and paired-end reads were generated.

#### Quality Control and Comparative Analysis

Raw data (raw reads) of fastq format were first processed through in-house perl scripts. In this step, clean data (clean reads) were obtained by removing reads containing adapter, reads containing ploy-N, and low-quality reads from raw data. At the same time, Q20, Q30, Guanine and Cytosine content (GC-content), and sequence duplication levels of the clean data were calculated. All the downstream analyses were based on clean data with high quality. The adaptor sequences and low-quality sequence reads were removed from the data sets. Raw sequences were transformed into clean reads after data processing. These clean reads were then mapped to the reference genome sequence. Only reads with a perfect match or one mismatch were further analyzed and annotated based on the reference genome. HISAT2 tool soft was used to map with the reference genome.

#### Gene Functional Annotation

Gene function was annotated based on the following databases: Nr (National Center for Biotechnology Information [NCBI] nonredundant protein sequences); Nr (NCBI nonredundant nucleotide sequences); protein family (Pfam); clusters of orthologous groups of proteins (KOG/COG); Swiss-Prot (a manually annotated and reviewed protein sequence database); Kyoto Encyclopedia of Genes and Genomes (KEGG) ortholog (KO) database; and gene ontology (GO).

#### Quantification of Gene Expression Levels

Quantification of gene expression levels was estimated by fragments per kilobase of transcript per million fragments mapped. The formula of FPKM is shown as follow:


$$\begin{array}{l} \text{FPKM}=\frac{\text{cDNA Fragments}}{\text{Mapped Fragments }\left(\text{Millions}\right)\times \text{Transcript length}\left(\text{kb}\right)} \end{array}$$

#### Differential Expression Analysis

Differential expression analysis of two conditions/groups was performed using the DESeq2_EBSeq. DESeq2 provides statistical routines for determining differential expression in digital gene expression data using a model based on the negative binomial distribution. The corrected *P*-values from this method accounted for multiple tests used by the key factor, which was false discovery rate (FDR). FDR < 0.01 and |log2 [fold change (FC)]|≥4 were set as the thresholds for differential gene expression.

#### GO and KEGG Pathway Enrichment Analysis

Gene ontology enrichment analysis of the differentially expressed genes (DEGs) was implemented by the GOseq R packages-based Wallenius noncentral hypergeometric distribution (Young et al., 2010), which can adjust for gene length bias in DEGs. KEGG (Minoru et al., 2008) is a database resource for understanding high-level functions and utilities of the biological system, such as the cell, the organism, and the ecosystem from molecular-level information, especially large-scale molecular datasets generated by genome sequencing and other high-throughput experimental technologies (http://www.genome.jp/kegg/). We used KOBAS software to test the statistical enrichment of differential expression genes in KEGG pathways (Mao et al., 2005).

### Protein-Protein Interaction

Sequences of DEGs were blasted (blastx) to the genome of a representative specie to get predicted protein protein interaction (PPI), which was existed in the STRING database (http://string-db.org/) to get the predicted PPI of these DEGs. Then the PPI of these DEGs was visualized in Cytoscape (Shannon, 2003).

### Enzyme Activity Assays

The details of reactive oxygen species (ROS) content determination and antioxidant enzyme activity in anthers were described previously (Liu et al., 2018b).

### Extraction of Total RNA, Primer Design, and Quantitative Real-Time PCR (qRT-PCR) Analysis

Extraction of total RNA from collected anther samples frozen at −80°C refrigerator, synthesis of cDNA, and the quantitative real-time (qRT-PCR) were performed as previously (Liu et al., 2018a) described. Specific primers were designed using the primer premier 5.0 software for qRT-PCR; the primer sequence details are provided in Supplementary Table 1.

## Results

### Phenotypic and Histological Differences Between Normal and HT Wheat

**Figure 1:**
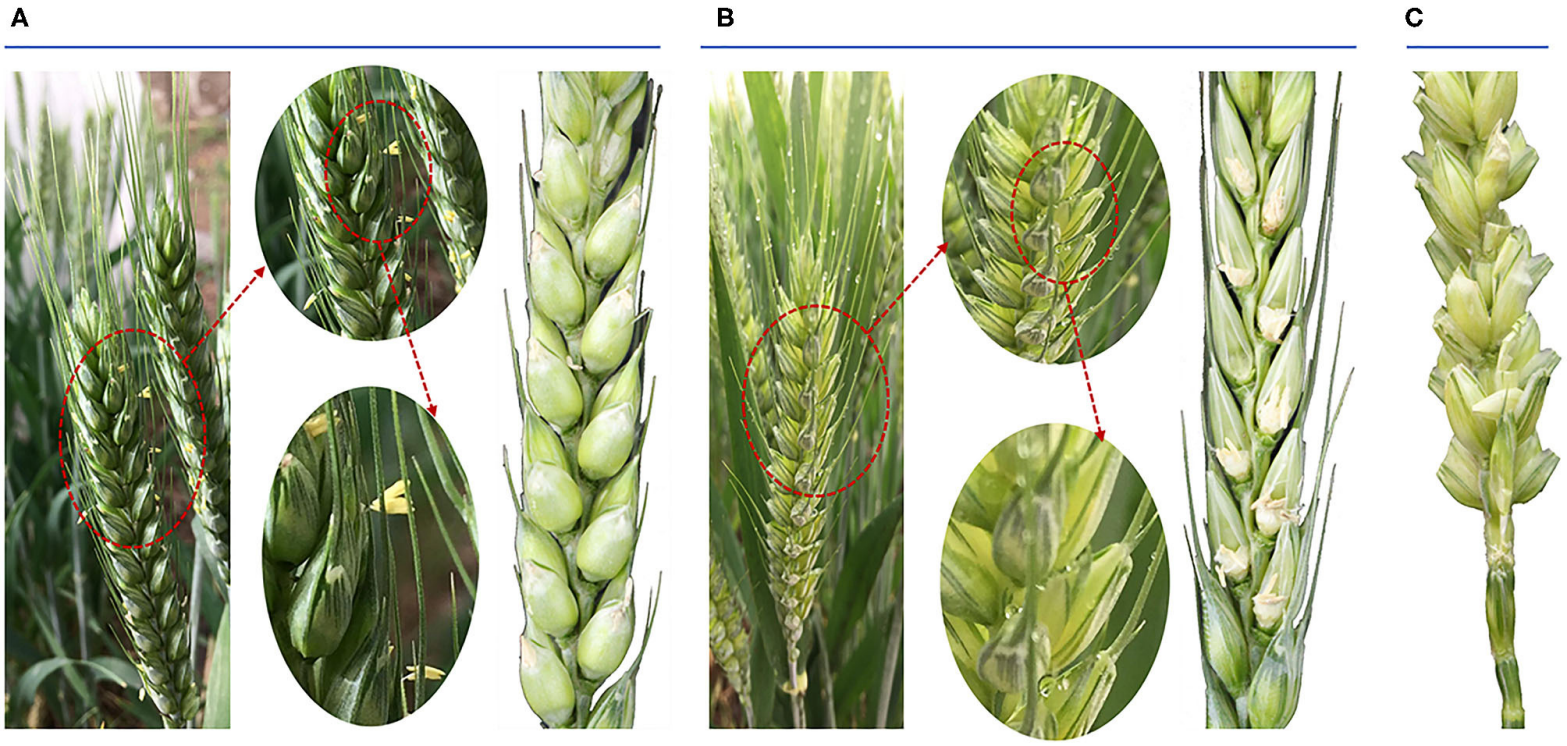
Phenotypic traits of both the anthesis period of wheat ear and the seed status after wheat pollination. **(A)** The morphology of Normal anthers in anthesis on spikelets and the seed status. **(B)** The morphology of high temperature-male sterile (HT-ms) anthers in anthesis on spikelets and the seed status. **(C)** The seed status after artificial pollination of HT-ms plant.

The phenotype of the anthesis period of wheat ear and seed status after wheat pollination between Normal and HT plants were compared. There were two marked differences in the wheat ear during the anthesis period. One was that the Normal wheat was greener than the HT-induced wheat; the other was that the Normal wheat anthers extended beyond the glume and the HT-induced wheat did not exhibit this phenomenon (Figures 1A,B). Before anthesis, we randomly bagged 40 HT-treated spikelets and 20 Normal plants. Finally, we got 20 Normal spikelets, 20 HT-male sterility spikelets, and 17 spikelets after artificial pollination (there were three spikelets that did not grow normally after the stem was broken). By selfing *via* sulfur paper bags, the results of seed setting rate showed that the Normal wheat reached 100% and the HT-induced wheat reached 0% (no seed). To prove that the occurrence of this phenomenon was caused by anther sterility, we conducted artificial pollination of the HT-induced wheat, and the results showed that the seed setting rate could reach more than 93% through artificial pollination (Figure 1C).

**Figure 2:**
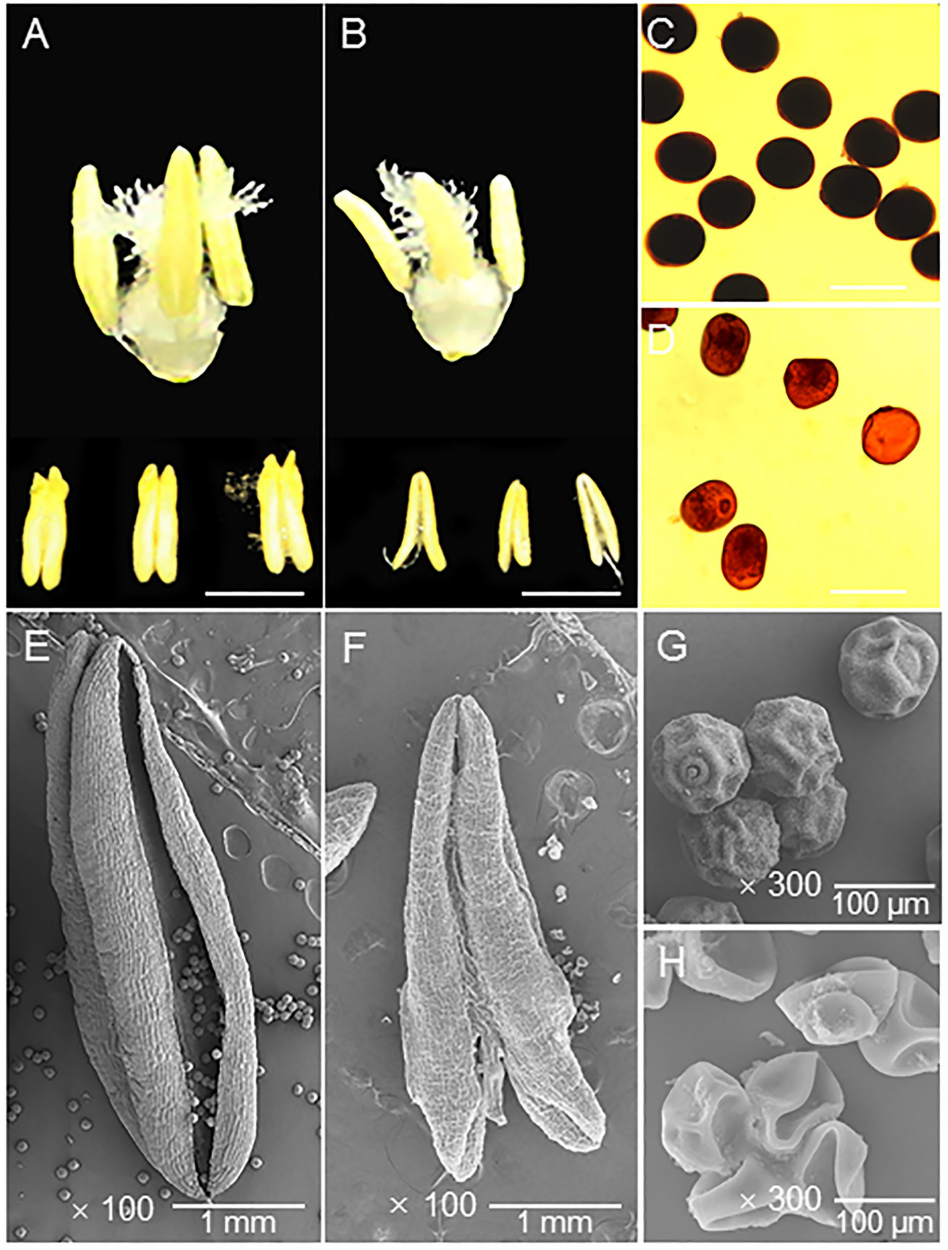
Appearance of the trinuclear stage anther and pollen grain in Normal and HT-ms plant. **(A)** The morphology of Normal trinuclear stage anthers. **(B)** The morphology of HT-ms trinuclear stage anthers. **(C)** Pollen grains from a Normal plant stained with potassium iodide iodine (KI-I_2_) solution. **(D)** I_2_-KI-stained pollen grains from an HT-ms plant. **(E,F)** The outermost surface of the epidermis of Normal **(E)** and HT-ms **(F)** anthers under scanning electron microscopy (SEM). **(G,H)** SEM analysis of pollen grains from Normal **(G)** and HT-ms **(H)** plants. Bars = 2 mm in **A,B**, 100 μm in **C,D**, 1 mm in **E,F**, and 100 μm in **G,H**.

From the anther phenotype comparison, the first obvious difference was that the HT-ms anthers were smaller than the Normal anthers (Figures 2A,B). In detail, the average length and width of 10 Normal anthers (randomly selected) were 3.71 and 1.27 mm, respectively (Supplementary Figure 2A); but the HT-ms anthers were 2.93 and 0.85 mm, respectively (Supplementary Figure 2B). Observation of the starch accumulation of mature pollen grains, using I_2_-KI solution staining, revealed that the starch accumulation of pollen grains was full and exhibited a completely black color after staining (Figure 2C). In contrast, pollen grains of the HT-ms anthers appeared light yellow after staining, which suggested little or no accumulation of starch (Figure 2D).

Moreover, we further analyzed the ultrastructural characteristics of these anthers and pollen grains by SEM. The Normal anther was larger than the HT-ms anther and had obvious dehiscence (Figures 2E,F). The pollen shape of the HT-ms anther appeared severely malformed, whereas the Normal pollen grains had particulate exine patterning and a nearly round shape (Figures 2G,H). These findings agree with the I_2_-KI staining results and indicate that the development of the anthers and pollen grains was defective in the HT-ms plants.

### RNA-Sequencing and Assessment of the Sequencing Results

The total RNA of anthers from these Normal and HT plants was sequenced using sequencing by synthesis technology with an Illumina system. We performed transcriptomic analysis of the two samples (Normal anthers of trinuclear stage and HT-ms anthers of trinuclear stage), with three biological replicates for each sample (named N1, N2, N3 and HT1, HT2, HT3, respectively), to profile the male sterile anthers response to HT stress. After sequencing quality control, the RNA-sequencing (RNA-seq) analysis obtained 54.74 Gb of clean data including three biological replicates in total, and the Q20 and the Q30 base percentage of each sample was not less than 95.82 and 90.17%, respectively (Supplementary Table 2). The clean reads of each sample were sequence-aligned with the designated reference genome (IWGS_RefSeq_assembly v1.0), and the efficiency of the alignment varied from 90.79 to 91.94% (Supplementary Table 3). A total of 136,235 unigenes were obtained after assembly using the HISAT2 software. We performed the functional annotation of the unigenes in various databases, including COG, KOG, GO, NR, Swiss-Prot, KEGG, eggNOG, and Pfam databases, which resulted in 126,335 unigenes successfully annotated. Of these unigenes, 46,937 were 300–1,000 bp in length, and 73,764 unigenes had a length of more than 1 kb (Table 1). Based on the alignment results, variable splicing prediction analysis, gene structure optimization analysis, and new gene discovery were performed, and 25,445 new genes were discovered, of which 16,172 were functionally annotated.

**Table 1:** Functional annotation statistics of the unigenes.

**Annotation database**	**Annotated number**	**300 ≤ length <1,000**	**Length ≥ 1,000**
COG_Annotation	35,213	9,269	25,496
GO_Annotation	84,177	28,470	52952
KEGG_Annotation	35,770	12,023	22,743
KOG_Annotation	55,816	16,537	38,306
Pfam_Annotation	92,375	30,056	60,082
Swiss Prot_Annotation	75,817	23,472	50,456
eggNOG_Annotation	113,772	40,158	69,405
NR_Annotation	126,052	46,784	73,669
All_Annotated	126,335	46,937	73,764

The Pearson's correlation coefficients of three replicates of Normal and HT-ms anthers showed that the sequencing data were highly repetitive (Supplementary Figure 3A). Gene expression levels were estimated with the FPKM values ranging from 0 to 3258.2 (Supplementary Figure 3B). Principal component analysis (PCA) confirmed that the Normal and HT-ms anthers were genetically distinct based on gene expression. The results of PCA showed that the explained values of PC1 and PC2 were 87.5 and 6.5%, respectively (Supplementary Figure 3C). The species distribution of the annotated unigenes is shown in Supplementary Figure 3D. DEGs were identified based on their expression levels in different samples, and functional annotation and enrichment analysis were performed.

### Identification and Annotation of DEGs

**Figure 3:**
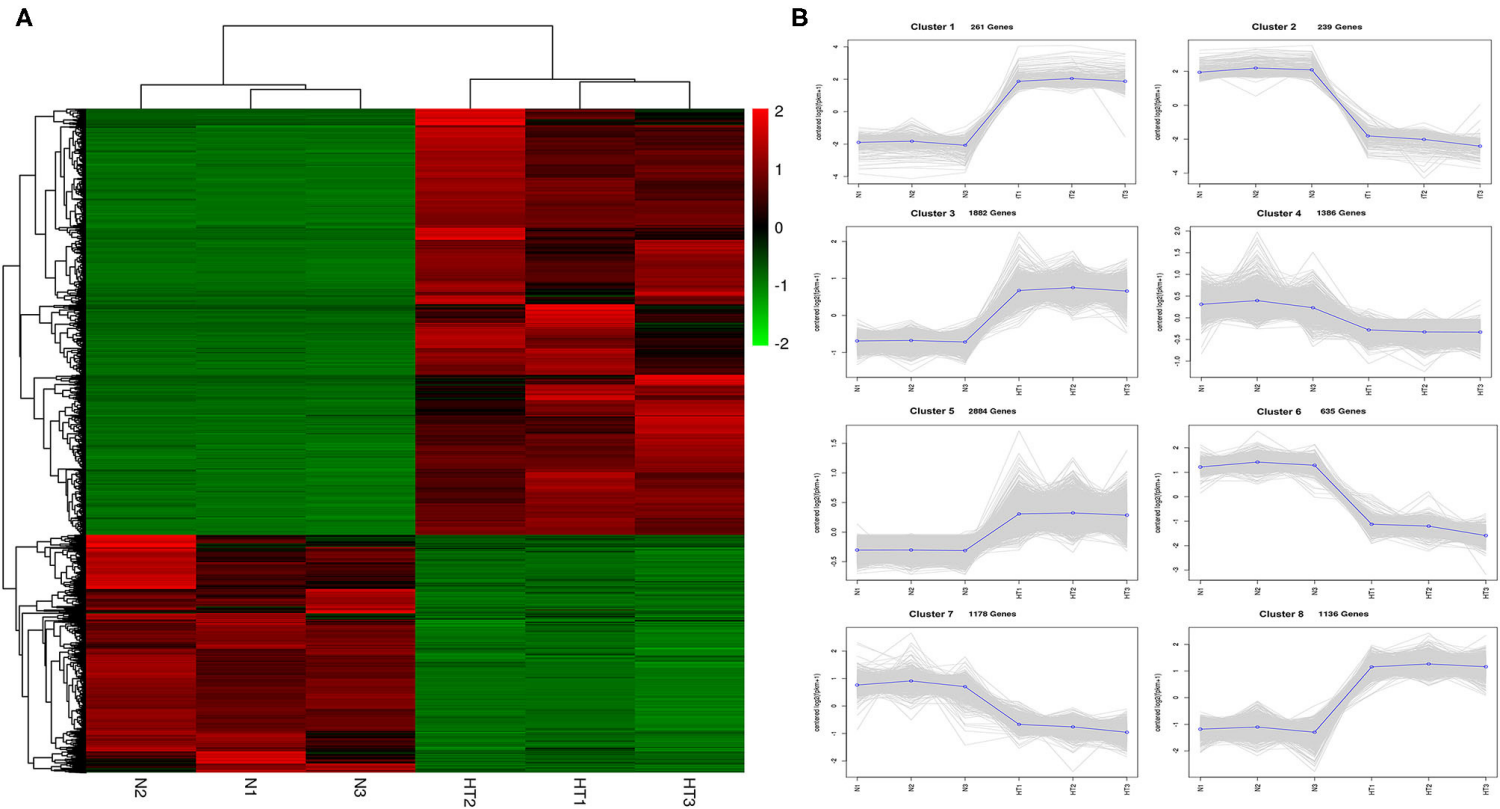
Gene expression profiles from Normal and HT-ms anthers. **(A)** Heat map diagrams representation of gene expression fold changes (FCs) triggered by the Normal and HT-ms anthers. The colors green, black, and red indicates low, medium, and high expression patterns of genes, respectively. **(B)** K-mean clustering of the DEGs in eight clusters between the Normal and HT-ms anthers. Thin gray lines represent the expression levels of individual genes. Thick blue lines represent the average expression level of genes in the cluster. The number of genes in each cluster is indicated.

**Figure 4:**
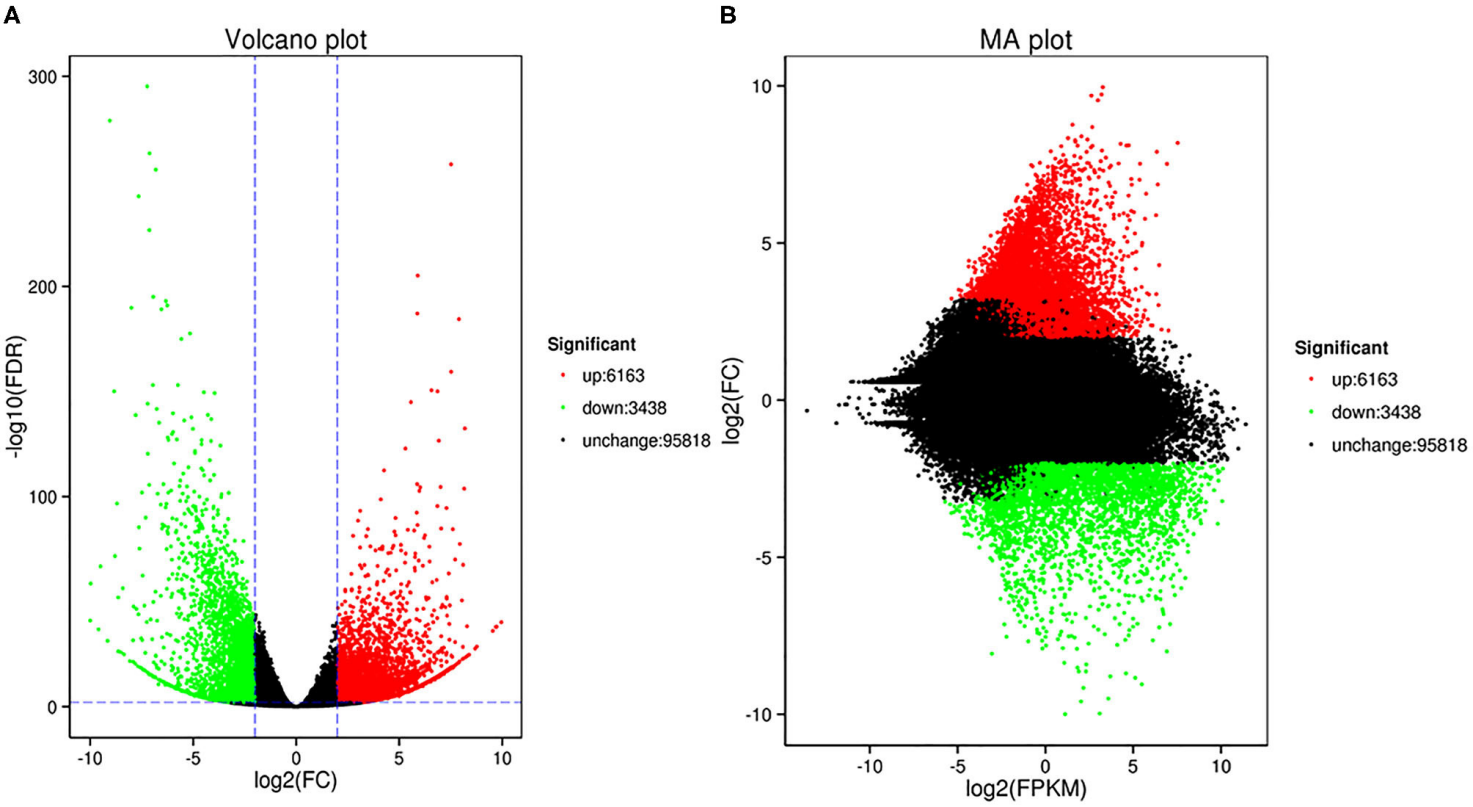
The volcano plot and MA plot show the changes in the abundance of unigenes between fertile anthers and HT-ms anthers. Each dot in the volcano map represents a gene. Green dots indicate significantly downregulated DEGs, red dots indicate significantly upregulated DEGs, and black dots represent non-DEGs. **(A)** Volcano plot; the abscissa represents the log 2 (FC) in the expression of a certain gene in two samples; the ordinate represents the negative log10 (false discovery rate [FDR]) in gene expression. **(B)** The abscissa is the A value: log2 (FPKM), which is the logarithmic value of the mean value of the expression in the two samples; the ordinate is the M value: log2 (FC), which is the logarithm of the fold difference in gene expression between the two samples.

In this study, we identified 9601 DEGs using Deseq2 software with parameters FC greater than or equal to 4 and an FDR correction set less than 0.01. To investigate the expression patterns of DEGs between the Normal anthers and HT-ms anthers, the gene expression levels were quantified with the FPKM method, and the gene expression profile clustering was subjected to the first analysis. Hierarchical clustering revealed the DEGs of Normal and HT-ms anthers based on their expression trends (Figure 3A). These genes were divided into two groups: one was upregulated and the other was downregulated. To explore the possible functions of the DEGs in more detail, k-means clustering analysis was used to further divide the DEGs into eight clusters with similar expressions between the Normal and HT-ms anthers (Figure 3B). Clusters 1, 3, 5, and 8 showed an upregulated expression trend with 261, 1,882, 2,884, and 1,136 genes, respectively. Clusters 2, 4, 6, and 7 showed a downregulated expression trend with 239, 1,386, 635, and 1,176 genes, respectively. Similarly, the map of volcanoes and Minus-versus-Add (MA) plots showed the same upregulation and downregulation of the expression results of the summation of these DEGs. Among them, the red dots show upregulated genes, totaling 6,163; the green dots show downregulated genes, totaling 3,438 (Figures 4A,B).

### GO Analysis of DEGs

The DEGs shared by the Normal anthers and the HT-ms anthers were divided into cellular components, molecular function, and biological processes. These three main categories were composed of 52 functional groups using GO assignment. As shown in Supplementary Figure 4, the cellular component category contained 15 functional groups, in which “cell part,” “cell,” “organelle,” and “membrane” were the top four represented terms, including 2,864, 2,857, 2,283, and 1,358 genes, respectively (Supplementary File 1). The top four significant GO nodes enriched to the DEGs were the external side of the cytoplasmic membrane-bounded vesicle (GO:0016023, Kolmogorov-Smirnov [KS]<1e-30), nucleosome (GO:0000786, KS<1e-30), plasma membrane (GO:0005886,KS <1.90E-13), and intracellular membrane-bounded organelle (GO:0043231, KS<3.00E-10), including 888, 185, 478, and 2,889 DEGs, respectively (Supplementary Table 4).

In the biological process category, the majority of functional groups were “metabolic process,” “cellular process,” “single-organism process,” and “response to stimulus” including 2,505, 2,494, 1,725, and 951 genes, respectively (Supplementary Figure 4). The top four significant GO nodes enriched to the DEGs were the external side of nucleosome assembly (GO:0006334, KS <1e-30), response to salt stress (GO:0009651, 2.80E-10), response to cadmium ion (GO:0046686, 1.10E-09), and DNA replication initiation (GO:0006270, 1.30E-09), including 182, 80, 62, and 38 DEGs, respectively (Supplementary Table 5).

In the molecular function category, the dominant function types were “binding,” “catalytic activity,” “transporter activity,” and “electron carrier activity,” including 2,645, 2,450, 320, and 129 genes, respectively (Supplementary Figure 4). The top four significant GO nodes enriched to the DEGs were the external side of protein heterodimerization activity (GO:0046982, 5.70E-26), peroxidase activity (GO:0004601, 2.50E-13), heme binding (GO:0020037, 4.10E-13), and aspartyl esterase activity (GO:0045330, 5.40E-11), including 179, 51, 126, and 25 genes, respectively (Supplementary Table 6).

**Figure 5:**
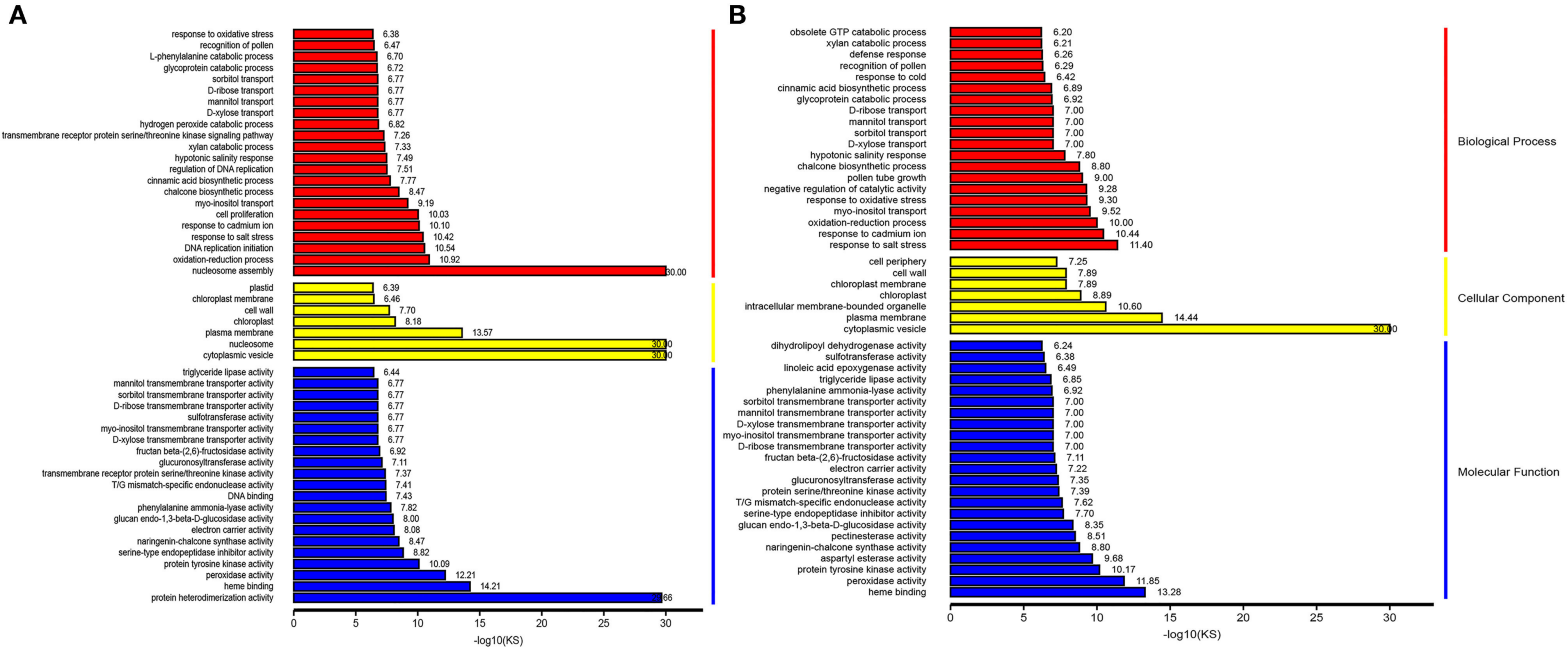
Representative images showing top enrichment gene ontology (GO) terms of the differentially expressed gene (DEGs). Red color, yellow color, and blue color indicates a biological process, cellular component, and molecular function, respectively. **(A)** Upregulated DEGs in Normal and HT-ms anthers. **(B)** Downregulated DEGs in Normal and HT-ms anthers.

To further identify the statistically up or downregulated categories of cellular components, molecular functions, and biological pathways related to male sterility induced by HT, we focused on the GO enrichment of these up or downregulated genes. Compared with the Normal anthers, the GO enrichment results of the upregulated genes reveal that the high-enriched biological processes are “nucleosome assembly,” “oxidation-reduction process,” “DNA replication initiation,” “response to salt stress,” and “response to cadmium ion” (Figure 5A). In this category, the downregulated genes are highly enriched “response to salt stress,” “response to cadmium ion,” “oxidation-reduction process,” “myo-inositol transport,” and “response to oxidative stress” (Figure 5B). Meanwhile, the GO enrichment results of the upregulated genes show that the high-enriched cellular component categories are “cytoplasmic vesicle,” “nucleosome,” “plasma membrane,” “chloroplast,” and “cell wall” (Figure 5A); and downregulated genes enrichment results in this category reveal that “cytoplasmic vesicle,” “plasma membrane,” “intracellular membrane-bounded organelle,” “chloroplast,” and “chloroplast membrane” are highly enriched (Figure 5B). In the molecular function category, the top five significant GO nodes enriched to the upregulated genes are the external side of “protein heterodimerization activity,” “heme binding,” “peroxidase activity,” “protein tyrosine kinase activity,” and “serine-type endopeptidase inhibitor activity” (Figure 5A). Meanwhile, the top five significant GO nodes enriched to the downregulated genes are the external side of “heme binding,” “peroxidase activity,” “protein tyrosine kinase activity,” “aspartyl esterase activity,” and “naringenin-chalcone synthase activity” (Figure 5B).

Furthermore, BiNGO software was used to analyze these genes. In the upregulated genes, besides the results mentioned above, the most interesting results were the enrichment of “response to reactive oxygen species,” “response to hydrogen peroxide,” “response to heat,” and “regulation of flower development” in the biological process category. In the downregulated genes, the most interesting results were the enrichment of “starch catabolic process,” “fatty acid biosynthetic process,” “response to jasmonic acid stimulus,” “phosphoinositide phosphorylation,” “positive regulation of GTPase activity,” and “activation of protein kinase C activity by G-protein coupled receptor protein signaling pathway” in the biological process category (Supplementary Files 2, 3).

### KEGG Analysis of DEGs

**Figure 6:**
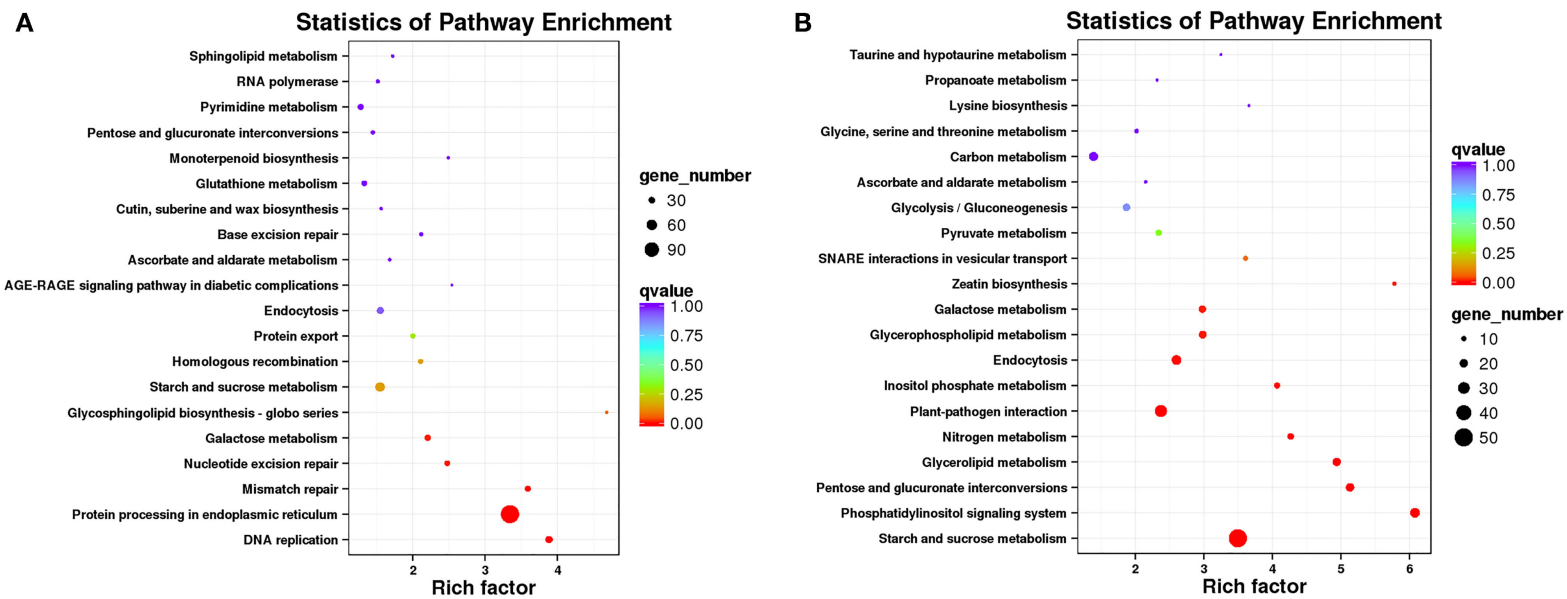
Top 20 significantly enriched Kyoto Encyclopedia of Genes and Genomes (KEGG) pathways. “Rich factor” indicates a high degree of enrichment. Dot size indicates how many DEGs are annotated to the pathway. The colors red, green, and blue indicate low, medium, and high expression patterns of genes, respectively. **(A)** Upregulated DEGs in Normal and HT-ms anthers. **(B)** Downregulated DEGs in Normal and HT-ms anthers.

The DEGs were annotated to 50 KEGG metabolic pathways in five categories, including cellular processes, environmental information processing, genetic information processing, metabolism, and organismal systems. The metabolic pathway composed of the most DEGs was “protein processing in endoplasmic reticulum” (ko04141), including 123 DEGs. The next metabolic pathways composed of more than 50 DEGs were “starch and sucrose metabolism” (ko00500), “protein processing in endoplasmic reticulum” (ko04141), “phenylpropanoid biosynthesis” (ko00940), “plant hormone signal transduction” (ko04075), “plant-pathogen interaction” (ko04626), and “endocytosis” (ko04144) (Supplementary Figure 5, Supplementary File 4). Interestingly, as shown in the Venn diagram, 17 genes were shared between the two pathways of “protein processing in endoplasmic reticulum” and “endocytosis.” Fifteen genes were the same in the metabolic pathways of “starch and sucrose metabolism” and phenylpropanoid biosynthesis. Three genes were shared between the two pathways of “protein processing in endoplasmic reticulum” and “plant-pathogen interaction.” Two genes were the same in the metabolic pathways of “plant hormone signal transduction” and “plant-pathogen interaction” (Supplementary Figure 6). Subsequently, we performed a KEGG enrichment analysis of up and downregulated genes. “Protein processing in endoplasmic reticulum” was the most abundant KEGG enrichment in upregulated DEGs (Figure 6A), and “starch and sucrose metabolism” was the most abundant KEGG enrichment in downregulated DEGs (Figure 6B). The most significant pathways of KEGG enrichment were the “glycosphingolipid biosynthesis-globo series” (ko00603; rich factor:4.68) and “phosphatidylinositol signaling system” (ko04070; rich factor:6.08) in upregulated and downregulated genes, respectively (Figures 6A,B and Supplementary File 4). Moreover, the PI signaling system and plant hormone signal transduction are both related to environmental information processing.

### DEGs Involved in Male Sterility in Wheat

The results of GO enrichment, BiNGO analysis, and KEGG analysis indicated that starch and sucrose metabolism, PI signaling system, heme binding, peroxidase activity, and response to oxidative stress are the most noticeable in the process of HT-sterility in wheat.

#### Further Analysis of the Crucial Pathway of Starch and Sucrose Metabolism

The most obvious characteristic of HT-ms pollen grains is less or even no starch accumulation, which has been clearly described in the I_2_-KI staining result and SEM result; the KEGG pathway of starch and sucrose metabolism was significantly enriched in the downregulated genes, and the enrichment factor reached 3.5. There were 50 DEGs, and the expression heat map is shown in Supplementary Figure 7. These downregulated genes may have significant effects on starch accumulation and glycometabolism.

#### Further Analysis of the Crucial Pathway of the PI Signaling System

The most significant pathway of KEGG enrichment was the “phosphatidylinositol signaling system” (rich factor 6.08) in downregulated genes (Figure 6B). In this pathway, 24 DEGs were enriched. What is interesting is that in BiNGO analysis, the pathways of “phosphoinositide phosphorylation” and “activation of protein kinase C activity by G-protein coupled receptor protein signaling pathway” were also enriched in downregulated genes. About 13 and 10 DEGs were enriched in these two pathways, respectively. Among these 23 genes, 20 genes are shared with the pathway of the “phosphatidylinositol signaling system” pathway.

#### Further Analysis of the Crucial Pathways of Heme Binding

As an important biomolecule, heme plays an important role in photosynthesis, respiration, electron transport, and other enzymatic reactions. In the GO enrichment pathway of molecular function, heme binding was enriched in both upregulated expression and downregulated expression, and the logarithm of the KS value was the largest in the downregulated DEGs (Figures 5A,B). In this pathway, the up and downregulated genes were 88 and 38, respectively.

#### Further Analysis of the Crucial Pathway of Peroxidase Activity and Response to Oxidative Stress

In this study, we found that the results of GO enrichment were closely related to peroxidase activity. The GO nodes of “peroxidase activity” (KS = 6.10e-13, 36 upregulated genes; KS = 1.40e-12, 15 downregulated genes), “response to oxidative stress” (KS = 4.20e-07, 76 upregulated genes; KS = 5.00e-10, 25 downregulated genes), and “hydrogen peroxide catabolic process” (KS = 1.50E-07, 11 upregulated genes) were significantly overrepresented in the DEGs (Supplementary File 5). Moreover, these 11 DEGs in the hydrogen peroxide catabolic process pathway were completely shared genes in the response to the oxidative stress pathway. In addition, 35 upregulated DEGs were enriched in the ROS pathway in BiNGO analysis.

**Figure 7:**
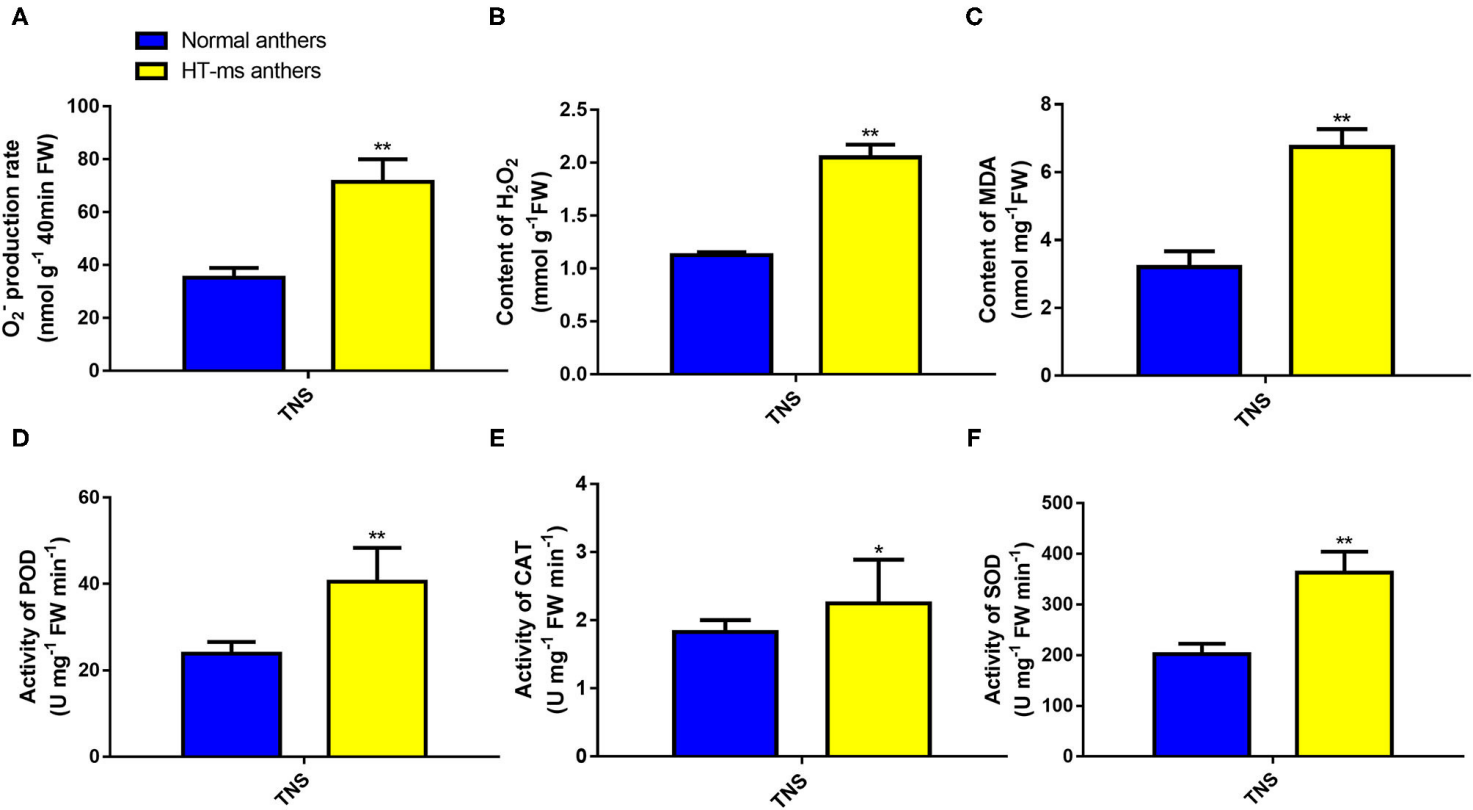
Reactive oxygen species (ROS) accumulation and POD, CAT, and SOD enzymatic activities in Normal and HT-ms anthers. The superoxide anion **(A)**, hydrogen peroxide **(B)**, and membrane lipid peroxidation (MDA) content **(C)** in Normal anthers and HT-ms anthers were measured at the trinuclear stage of development. The enzymatic actives of peroxidase **(D)**, catalase **(E)**, and superoxide dismutase **(F)** in Normal anthers and HT-ms anthers were also measured at the trinuclear stage. A single sample was used for three independent replicates (*n* = 3). *, ** =Significantly different from the Normal anthers control at *p* < 0.05 and *p* < 0.01, respectively.

**Figure 8:**
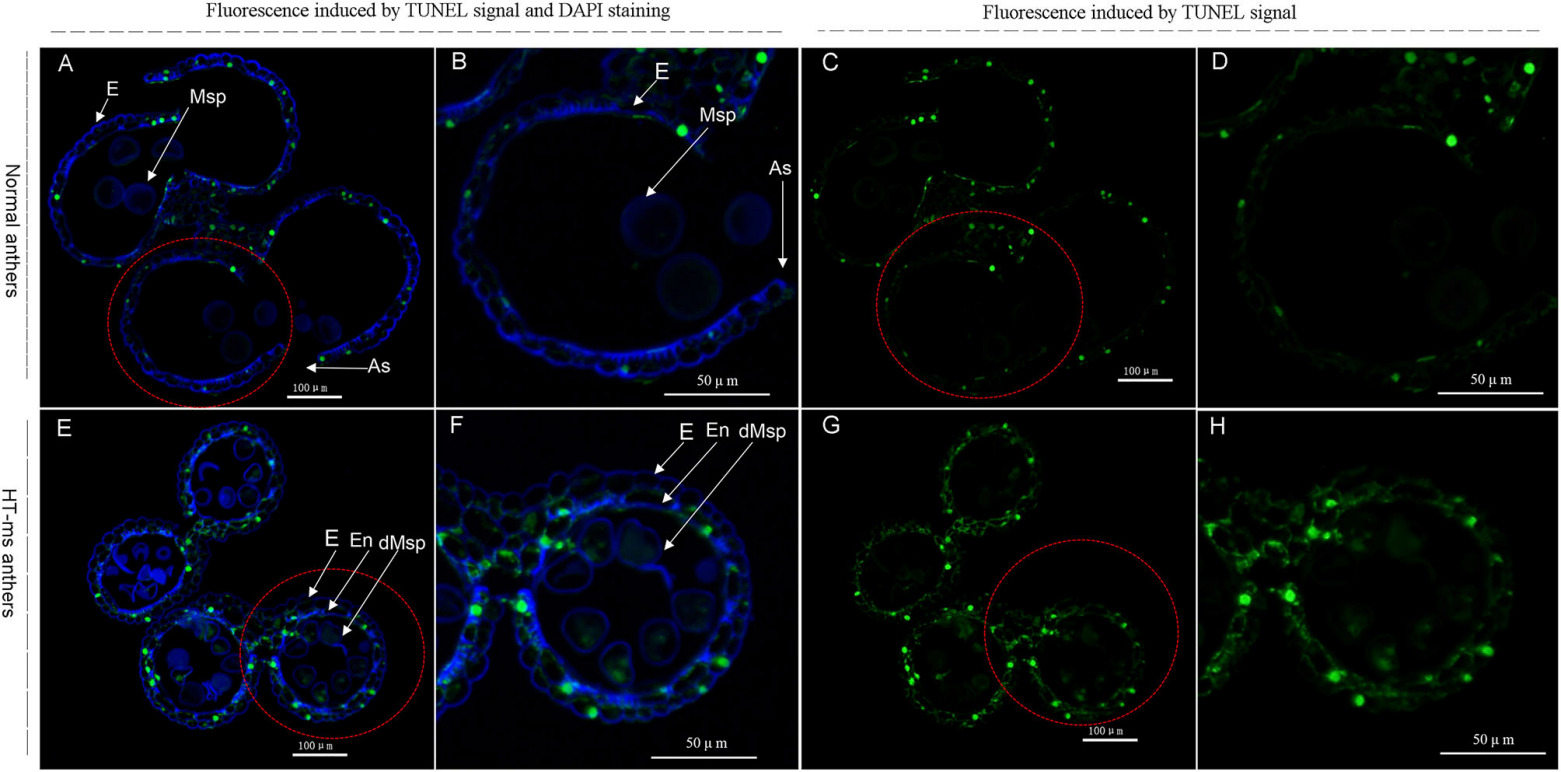
TUNEL assays to detect anther tapetum programmed cell death (PCD) in Normal anthers and HT-ms anthers at the trinucleate stage. As, anther slit; E, epidermis; En, endothecium; Msp: microspores. dMSP: degenerated microspore. The yellow-green fluorescence indicates nuclei with TUNEL-positive staining. The marked red dashed circle is the part that needs to be enlarged. **(A–D)** Normal anthers. **(E–H)** HT-ms anthers. Scale bars = 100 μm **(A,C,E,G)**; 50μm **(B,D,F,H)**.

Therefore, to confirm the change in expression of these DEGs and to correlate this change with enzyme activity, we determined the ROS levels, POD, and the other antioxidant activity, and performed a TUNEL assay for DNA damage. Compared with the Normal anthers, the rate of O^2−^ production was significantly higher in the HT-ms anthers in the trinuclear stage (Figure 7A). We were not surprised to find that the HT-ms anthers also possessed a greater H_2_O_2_ content in the trinuclear stage anthers, as excess O^2−^ was catalyzed to form H_2_O_2_ (Figure 7B). One of the hazards of the accumulation of ROS (O^2−^, H_2_O_2_) is the initiation or intensification of membrane lipid peroxidation (MDA), resulting in damage to the cell membrane system. Therefore, we measured MDA content, and the results showed that MDA levels were significantly higher in the HT-ms anthers than in the Normal anthers (Figure 7C). The results of antioxidative enzyme measurement showed that the activities of POD, superoxide dismutase (SOD), and catalase (CAT) in the HT-ms anthers were also significantly higher than those in the Normal anthers (Figures 7D–F). Then the question arose whether the increased antioxidant enzyme activity can scavenge the excess ROS so that it does not cause damage to the cells. With this question in mind, we performed TUNEL assays on anther sections. From the results, we found that the TUNEL fluorescence signal in the wall and microspore of the HT-ms anthers section was stronger than in the Normal anthers (Figures 8A–H). This also indicates that the increase in antioxidant enzyme activity did not save cells from being stressed by excessive ROS levels, resulting in aggravated programmed cell death (PCD).

### PPI Network Analysis of DEGs of the Crucial Pathway Involved in Wheat Male Sterility by Cytoscape Software

**Figure 9:**
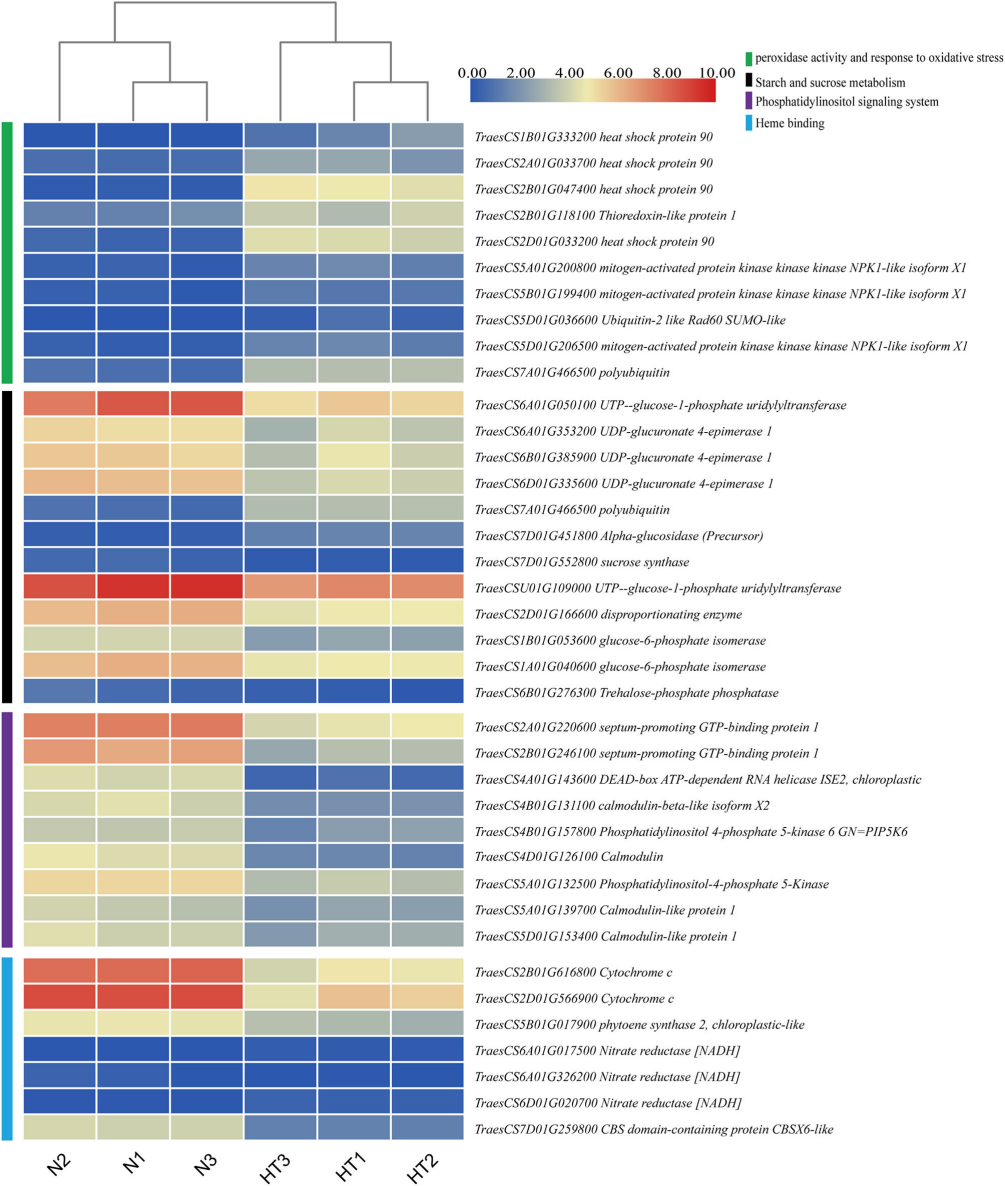
Hierarchical clustering analysis of hub DEGs in the crucial pathway. The green, black, purple, and cyan vertical boxes represent the peroxidase activity and response to oxidative stress pathway, starch and sucrose metabolism pathway, phosphatidylinositol (PI) signaling system, and heme binding, respectively. The gradient color of the legend represents the log2 (FPKM) value.

To explore and discover the hub genes in the four crucial pathways, we constructed a PPI network for each pathway with Cytoscape software (Supplementary File 5). According to the analysis results, we found 10 hub genes in the peroxidase activity and response to oxidative stress pathway. These genes are potentially annotated as HSP90, thioredoxin-like protein 1, mitogen-activated protein kinase (MAPK) kinase kinase NPK1-like isoform X1, Ubiquitin-2 like Rad60 SUMO-like protein, and polyubiquitin. Moreover, some proteins that are the first neighbors to thioredoxin-like protein 1 (*TraesCS2B01G118100*) involve peroxidase, calreticulin, and HSP (Supplementary Figure 8). We found 12 hub genes in the starch and sucrose metabolism pathway, which were related to UTP-glucose-1-phosphate uridylyltransferase, UDP-glucuronate 4-epimerase 1, alpha-glucosidase (precursor), sucrose synthase, disproportionating enzyme, glucose-6-phosphate isomerase, trehalose-phosphate phosphatase, and polyubiquitin. There were nine hub genes, involving septum-promoting guanosine triphosphate (GTP)-binding protein 1, DEAD-box ATP-dependent RNA helicase ISE2, calmodulin (CaM), and phosphatidylinositol-4-phosphate 5-Kinase (PIP5K) in the PI signaling system pathway. In addition, there were seven hub genes in the pathway of heme binding, which were related to cytochrome c, phytoene synthase 2, nitrate reductase [NADH], and CBS domain-containing protein CBSX6-like. The expression of these hub genes is shown in Figure 9 as a heat map according to the log value of the FPKM.

### Validation of the RNA-Seq Data

**Figure 10:**
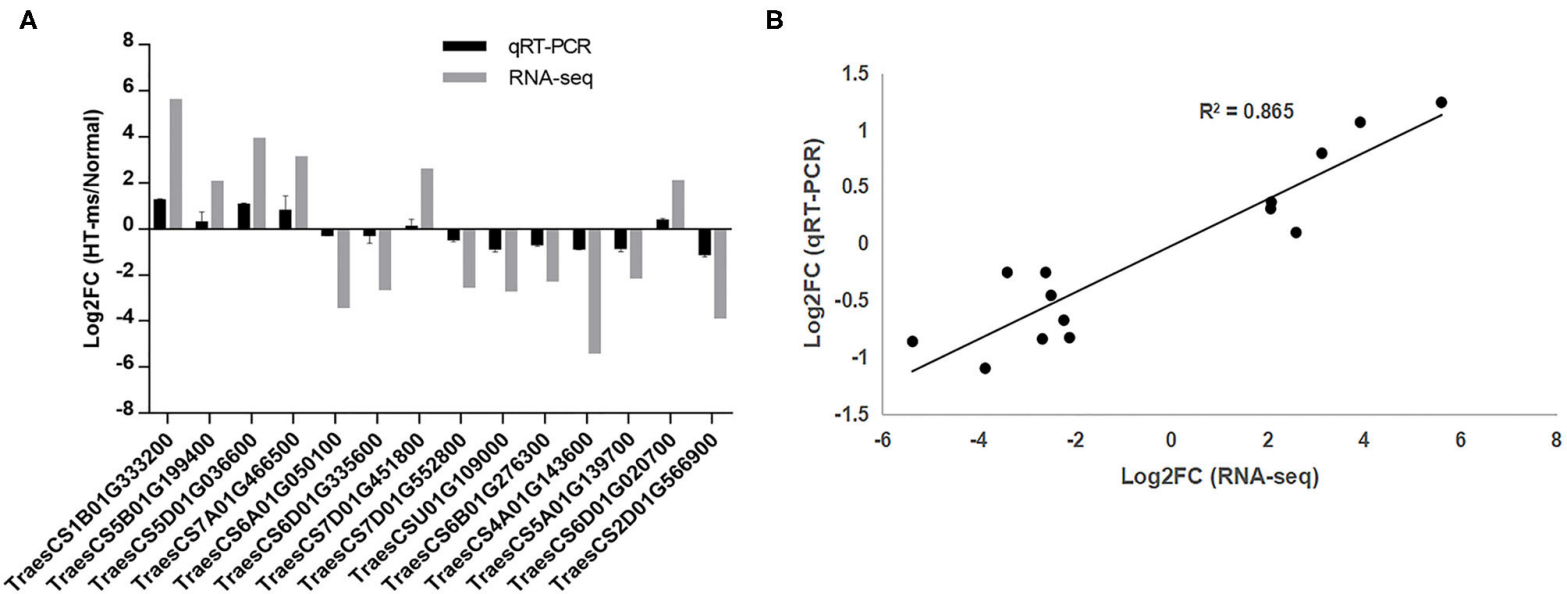
Quantitative real-time PCR (qRT-PCR) validation of the RNA sequencing results for some hub DEGs. **(A)** Log2 (FC) represents the logarithm of the FC in expression for HT-ms anthers relative to Normal anthers. **(B)** Correlation analysis of hub DEGs between qRT-PCR and RNA-seq data. The scatter plot indicates the log2-transformed gene expression values in qRT-PCR and RNA-seq.

To verify whether our RNA-seq data output represented the true variation of the transcripts, the expression levels of 14 hub DEGs were randomly selected from all the hub DEGs and examined by qRT-PCR experiment. The primer sequence details are provided in Supplementary Table 1. Further qPCR results showed similar expressive tendencies to those obtained from the RNA sequencing (Figure 10A). The results of qRT-PCR and RNA sequencing showed a high agreement with each other. A strongly positive correlation (*R*^2^= 0.865) of linear regression analysis between qRT-PCR and RNA-seq suggested that the RNA-seq results in the present work are accurate and dependable (Figure 10B).

## Discussions

In this study, using the RNA-seq technology, transcriptome changes of wheat anthers were obtained under both normal and HT conditions. In total, the RNA-seq analysis obtained 54.74 Gb clean data including three biological replicates after sequencing quality control. Gene functional annotation toward various databases (COG, KOG, GO, NR, Swiss-Prot, KEGG, eggNOG, and Pfam) was conducted after reads assembly. A total of 9601 DEGs were defined by a strict criterion and the calculation of gene expression for each unigene. According to the results of GO enrichment, KEGG enrichment, and BinGO analysis, we focused on pathways that were the most noticeable in the process of HT sterility, including starch and sucrose metabolism, peroxidase activity, and response to oxidative stress, PI signaling system, and heme binding. Moreover, hub DEGs involved in these pathways and q-RT PCR verification analysis are also discussed below.

### Hub-DEGs Related to Starch and Sucrose Metabolism

Compared with the Normal anthers, in the process of male sterility caused by HT, the results of I_2_-KI staining and SEM showed that the most obvious morphologies of sterile anthers were thinness and indehiscence. The sterile pollen grains had almost no starch accumulation and appeared severely malformed under SEM. In the KEGG enrichment results of downregulated DEGs, starch and sucrose metabolism was the most enriched pathway (Figure 6B), which also verified those phenotypes exhibited by sterile anthers. As a key enzyme in carbohydrate metabolism, UTP-glucose-1-phosphate uridylyltransferase (*TraesCSU01G109000*) is usually also called UDP glucose pyrophosphorylase (UGPase) that catalyzes the reversible production of glucose-1-phosphate and UTP to UDP-glucose and pyrophosphate (Schomburg and Stephan, 1997; Chen et al., 2007). Studies in rice have shown that silencing the Ugp1 gene can lead to the complete sterility of anthers. During the development of anthers from the uninucleate stage to the trinucleate stage, the pollen grains of plant anthers with gene silencing are completely degenerated, and only some remnants remain in the anther locules, while the tapetum layer becomes very large with cavitation (Chen et al., 2007). In addition, a recent study has shown that UGPase is a key candidate gene for male sterility in isonuclear alloplasmic male-sterile lines of wheat (Liu et al., 2020). The results of our transcriptome and qRT-PCR indicate that this gene is significantly downregulated in sterile anthers (Figure 10A). KEGG_map (ko00500) shows that UGPase is the most important enzyme from α-D-glucose-1p to UDP-glucose (2.7.7.9), and its downregulation may result in a decrease in UDP-glucose, which will lead to an insufficient supply of sucrose (Supplementary Figure 9). Moreover, sucrose synthase (*TraesCS7D01G552800*), which catalyzes the reversible reaction of sucrose synthesis and decomposition, is also downregulated (Figure 10A). UDP-glucuronate 4-epimerase (*TraesCS6D01G335600*) belongs to the isomerase family and is involved in starch and sucrose metabolism and nucleotide sugar metabolism. The gene expression that regulates this enzyme has a significant downward trend in our transcriptome and qRT-PCR results (Figure 10A). As an essential enzyme of catabolic glycolysis and anabolic gluconeogenesis, glucose-6-phosphate isomerase catalyzes the reversible isomerization of glucose 6-phosphate and D-fructofuranose 6-phosphate (Petersen, 2007). Our results suggest that the enzyme is downregulated and may play an important role in the process of HT-induced male sterility in wheat (Figure 10). In addition, in this pathway, one of the hub genes we found was a polyubiquitination gene (*TraesCS7A01G466500*). The first linked genes of this polyubiquitination gene are mainly glucose-6-phosphate isomerase and alpha-glucan phosphorylase. This gene is significantly upregulated, which may be related to the ubiquitination and degradation of these two enzymes (Supplementary File 5).

### Hub DEGs Related to Peroxidase Activity and Response to Oxidative Stress

Interestingly, the polyubiquitination gene mentioned above is also one of the hub genes of the pathway of peroxidase activity and response to oxidative stress (Supplementary File 5). Oxidative stress refers to the excessive production of highly active molecules such as ROS when the organism is subjected to various harmful stimuli, and the degree of oxidation exceeds the removal of oxides, leading to damage to cellular components. In wheat, male sterile anthers caused by chemical hybridization agents have abnormal levels of ROS, which may affect the biosynthesis and degradation of tapetum cells and cell walls (Liu et al., 2018b). During anther development, ROS levels are closely related to the correct time of PCD in tapetum (Durme and Nowack, 2016). Studies have shown that the tapetum cells provide building materials for the pollen wall to support pollen development through PCD (Chang et al., 2011). NADPH oxidase is tissue-specific in *Arabidopsis* and can regulate H_2_O_2_ level, which in turn promotes PCD of tapetum cells (Durme and Nowack, 2016). Our results show that the ROS levels of HT-sterile anthers, including the rate of O^2−^ production, H_2_O_2_ content, and MDA content, are significantly higher than in the Normal anthers (Figures 7A–C). At the same time, the antioxidant enzymes used to scavenge active oxygen, including SOD, CAT, and POD enzymes, also show a significant increase in sterile anthers (Figures 7D–F). Similarly, the study of Aegilops uniaristata cytoplasmic male sterility (CMS) wheat also showed that the activities of SOD and POD of sterile anthers were higher than those of maintainer anthers throughout the anther development to prevent excessive accumulation of ROS (Liu et al., 2018d). The accumulation of ROS increases, and at the same time antioxidant enzymes also increase, so it is impossible to confirm whether anthers are really affected by ROS during sterility. In view of this, we conducted a TUNEL assay, and the results showed that the ROS accumulation signal of sterile anthers was significantly stronger than that of Normal anthers, which indicated that the ROS did have an important influence on the HT sterility process of anthers (Figure 8). Previous studies have shown that the level of ROS and the contents of SOD, POD, and CAT were significantly increased, accompanied by a content increase in HSP70, HSP90, HSP17, and HSP26 after high-temperature treatment of wheat pollen (Kumar and Goswami, 2014). Moreover, HSPs are molecular chaperones essential for the maintenance and/or restoration of protein homeostasis. In the study of transcriptome analysis of heat susceptible wheat leaves and heat tolerant wheat leaves, the researchers found that 117 probe sets encoding various HSPs were upregulated by the heat treatments, with the highest FC 11.8 times (Qin et al., 2008). Transcriptome analysis of HT stress during grain filling shows that small HSPs and HSP70 play a crucial role in regulating heat stress tolerance (Rangan et al., 2019). In cotton, heat stress has been shown to increase the gene expression of HSP90 and other HSPs in pollen of heat-sensitive lines (Song et al., 2015). In our study, we found that the DEGs expression of HSP90 was significantly upregulated in HT-sterile anthers, which may be related to the process of anther sterility caused by HT stress (Figures 9, 10A, *TraesCS1B01G333200*).

In plants, thioredoxins are important regulators of thiol-disulfide status and play major roles in ROS processing and defense responses (Noctor et al., 2017). One of our hub DEGs is thioredoxin-like protein 1 (*TraesCS2B01G118100*), which is upregulated in sterile anthers (Figure 9), which may be related to the large accumulation of ROS in sterile anthers. In addition, in the animal kingdom, thioredoxin, if oxidized, dissociates from the apoptotic signal-regulating Kinase 1, thereby activating downstream c-jun n-terminal kinase and MAPK pathways to induce apoptosis (Mochida, 2000; Liu and Min, 2002). Several studies have demonstrated that the MAPK pathway is evolutionarily conserved no matter in the plant or animal kingdom (Ligterink and Hirt, 2001; Hao et al., 2015). It has been reported that MAPK cascade members act as critical regulators in mediating crop tolerance to abiotic stresses, possibly through regulation of the ROS metabolism (Hao et al., 2015). Interestingly, MAPK kinase kinase NPK1-like isoform X1 appeared in our hub DEGs (*TraesCS5B01G199400*) (Figures 9, 10A). This suggests that thioredoxin and MAPK pathways are not only associated with the accumulation of ROS but may play an important role in HT-induced male sterility in wheat.

### Hub-DEGs Related to the PI Signaling System

The PI signaling system plays a critical role in plant growth and development, involving cell differentiation and proliferation, gene expression, reproduction, aging, and cell response to environmental stress (Zhang et al., 2019; Liu et al., 2020). PI mainly consists of 1,2-diacylglycerol phosphate and inositol. It plays an important role in cell morphology, signal transduction, and various physiological functions of cells (Munnik and Vermeer, 2010). PIP5K is a key enzyme in the PI signaling system pathway and it participates in the vesicle transport of sucrose from the tapetum to the microspores. Thus, downregulation of the PIP5K gene (*TraesCS4B01G157800*) will affect the transport of sucrose to the microspores, thereby hindering the normal development of the pollen wall. This is consistent with our observation that HT-sterile anthers are thinner than Normal anthers. In addition, downregulation of diacylglycerol kinase (2.7.1.107) will affect the normal synthesis of phosphatidic acid (PA), which may eventually lead to a decrease in PI (Supplementary Figure 10). When cells are stimulated, PI 4, 5-bisphosphate produces two second messenger molecules, diglycerides, and inositol 1,4,5-triphosphate, which regulate the activity of protein kinase C and the concentration of intracellular Ca^2+^, respectively. Four of the hub DEGs in our results are CaM-related genes in the pathway of the PI signaling system (Figure 9). CaM can function alone, or it can combine with calcium ions to form a complex to interact with downstream target proteins and participate in a variety of cellular activities (Zeng et al., 2015). Tirlapur *et al*. suggested that calcium and CaM mainly accumulate in the tapetum and gradually transfer to the inner wall of anther chamber and during anther development, which may be related to pollen development and anther dehiscence (Tirlapur and Willemse, 1992). Our results show that the expression of DEGs that regulate CaM and CaM-like proteins are downregulated in sterile anthers, which may cause the signal transduction of calcium ions to be affected and is closely related to the occurrence of male sterility in wheat under HT stress. In rice, APOPTOSIS INHIBITOR5 (API5) interacts with two DEAD-box ATP-dependent RNA helicase, named AIP1 and AIP2. These two proteins can form dimers and directly interact with the rice cysteine protease gene. Suppression of AIP1/2 leads to the downregulation of the cysteine protease gene and then leads to male sterility, which is similar to the inhibition of the cysteine protease gene expression (Li et al., 2011). Our results also identified DEAD-box ATP-dependent RNA helicase (*TraesCS4A01G143600*), which showed a significant downregulation expression trend in sterile anthers (Figure 10A), indicating that DEAD-box ATP-dependent RNA helicase is involved in the process of pollen abortion caused by HT. Pollen development involves the dynamic changes of cytoplasmic and subcellular components, the cytoskeleton, and vacuole. Thus, the various components of the PI signaling system are involved in vacuolar changes and vesicle transport during pollen development (Krau and Haucke, 2007). Therefore, the downregulation of the hub DEGs related to the PI signal system may be a very important factor in the HT-sterility anthers.

### Hub DEGs Related to Heme Binding

Heme, one of the products of the tetrapyrrole biosynthesis pathway, can modulate this pathway by feedback inhibition and play a role in regulating PCD in plants (Chattopadhyay et al., 2015). Cytochrome c is important water-soluble redox hemoglobin in the living system. The whole molecule is composed of a peptide chain wrapped with a heme. Meanwhile, cytochrome c is one of the redox active molecules responsible for electron transfer in plant mitochondria, and its release is closely related to PCD (Martínez-Fábregas et al., 2014; Li et al., 2017; Tian et al., 2019). Most of the proteins related to CMS have a transmembrane structure, which can destroy the mitochondrial membrane structure or change the permeability of the mitochondrial membrane and induce the release of cytochrome c through the accumulation of a large amount of ROS and stimulate the abnormal occurrence of PCD in male reproductive tissues (Priyadarshan, 2019). In our study, the DEGs of cytochrome c (*TraesCS2B01G616800* and *TraesCS2D01G566900*) were downregulated in sterile anthers, which may be directly related to PCD and male sterility. In addition, phytoene synthase is an important enzyme in carotenoid biosynthesis. An important function of carotenoids is to inhibit the production of ROS and to remove ROS, protecting organisms from excessive accumulation of ROS (Shen et al., 2018). Based on this principle, we speculate that the hub DEG of phytoene synthase 2 (*TraesCS5B01G017900*) is downregulated in sterile anthers, which may be related to the excessive accumulation of ROS (Figure 9).

The cracking of angiosperms anthers is directly related to the endothecial secondary wall, which is mainly composed of lignin, and its lignification depends on the level of ROS in cells (Bonner and Dickinson, 1989; Kawasaki et al., 2006). The CBS domain-containing proteins are a large superfamily of proteins that conserve CBS domains, which play a regulatory role for many enzymes. It has been reported that CBSX1 and CBSX2 are related to the level of ROS activated by thioredoxin and directly involved in the deposition of lignin (Wook et al., 2013; Shin et al., 2020). Moreover, there is research evidence to confirm that CBSX3 knockdown plants are severely sterile, which is caused by the failure of anther dehiscence due to secondary wall thickening (Shin et al., 2020). In sterile wheat anthers caused by HT, the anther wall does not display dehiscence, and the expression of CBS domain-containing protein CBSX6-like (CBSX6-L) gene (*TraesCS7D01G259800*) is downregulated in sterile anthers (Figure 9). This is consistent with the sterility results of the CBSX3 knockdown in *Arabidopsis*, which also shows CBSX6-L plays an important role in the male sterility process caused by HT.

## Conclusions

In this study, we covered the wheat in the differentiation of stamen and pistils stage (Feekes growth stage 8.5) with transparent plastic film to cause HT stress, thereby constructing HT-induced male sterile plants. In order to explore the molecular mechanism and hub DEGs that cause HT male sterility in wheat, we conducted transcriptome analysis of Normal anthers and HT-ms anthers during the trinucleate stage. GO and KEGG pathway enrichment analysis revealed that these DEGs are involved in the starch and sucrose metabolism, PI signaling system, peroxidase activity and response to oxidative stress, and heme binding. Moreover, the results of TUNEL assays further confirmed the excessive accumulation of ROS in sterile anthers. In addition, 38 hub DEGs were obtained from the PPI network analysis of these pathways. These results provide a theoretical basis and technological approach for further study of the abortive interaction mechanism induced by HT in wheat.

## Data Availability Statement

The datasets presented in this study can be found in online repositories. The names of the repository/repositories and accession number(s) can be found below: NCBI SRA BioProject, accession no: PRJNA750863.

## Author Contributions

HL and GYi conceived and designed the experiments and wrote the manuscript. LH, CL, and XW collected and analyzed the phenotypic data. ZY, YH, GYa, and KM helped to collect anthers and improve the method and edited the manuscript. ZS participated in supervision and helped to revise the manuscript. All authors contributed to the article and approved the submitted version.

## Funding

We gratefully acknowledge the funding sources. This study was supported by the 2019 Postdoctoral Research Project Start-up Funding of Henan Province (No. 226152), the Department of Science and Technology Planning Project of Henan Province (Nos. 202102110173), the 2019 Young Master Teacher Funding Project of Zhoukou Normal University (No. ZKNU20190022), and the National Natural Science Foundation (No. 32071478).

## Conflict of Interest

The authors declare that the research was conducted in the absence of any commercial or financial relationships that could be construed as a potential conflict of interest.

## Publisher's Note

All claims expressed in this article are solely those of the authors and do not necessarily represent those of their affiliated organizations, or those of the publisher, the editors and the reviewers. Any product that may be evaluated in this article, or claim that may be made by its manufacturer, is not guaranteed or endorsed by the publisher.
