# Supplementary material for: Comparative Transcriptome Analysis of Male Sterile Anthers Induced by High Temperature in Wheat (Triticum aestivum L.)
Source: Front Plant Sci. 2021 Oct 25;12:727966. doi: 10.3389/fpls.2021.727966 (PMC8573241; doi:10.3389/fpls.2021.727966)
Supplement: Supplementary file 1 [file Table_1.docx]

Table S1 Target genes for analysis of qRT-PCR.

| Primer name | Primer sequences (5´–3´) | BP number |
| --- | --- | --- |
| TraesCS1B01G333200-F | 5´-GAAGCACGGCGTCGACATCT-3´ | 20 |
| TraesCS1B01G333200-R | 5´-CTTCGACATCGACAGCCTCG-3´ | 20 |
| TraesCS5B01G199400-F | 5´-GAGTTGATCAAACTTCCAATG-3´ | 21 |
| TraesCS5B01G199400-R | 5´-ATGGTATCACAAGGCGTTGC-3´ | 20 |
| TraesCS5D01G036600-F | 5´-CGTCGTCGGCTGAGGGAATG-3´ | 20 |
| TraesCS5D01G036600-R | 5´-ACAGACATCCTCTGCTGCAG-3´ | 20 |
| TraesCS7A01G466500-F | 5´-GAGTCTACTCTCCACTTGGT-3´ | 20 |
| TraesCS7A01G466500-R | 5´-GATGCCTAGAGAACACGATG-3´ | 20 |
| TraesCS6A01G050100-F | 5´-CAAGGGGAAGACTGACAAG-3´ | 19 |
| TraesCS6A01G050100-R | 5´-GTATTCTTTTCCCTGCGAGA-3´ | 20 |
| TraesCS6D01G335600-F | 5´-CTCTCCTACTACGGCTACAC-3´ | 20 |
| TraesCS6D01G335600-R | 5´-TGATCCGGGAGTGGTCGGAA-3´ | 20 |
| TraesCS7D01G451800-F | 5´-ATGATTGGCTCGGCTGGTCG-3´ | 20 |
| TraesCS7D01G451800-R | 5´-CAGTGTGCAGTGCAGCGCGA-3´ | 20 |
| TraesCS7D01G552800-F | 5´-GAAAGCCGCGGAACCGGATC-3´ | 20 |
| TraesCS7D01G552800-R | 5´-TCAGAACACAGTCATGGCTC-3´ | 20 |
| TraesCSU01G109000-F | 5´-CAAGCTCTCTCTCTCGCAAC-3´ | 20 |
| TraesCSU01G109000-R | 5´-GAGACGAGGCTGATGAACC-3´ | 19 |
| TraesCS6B01G276300-F | 5´-AAGTGATGGAGTTCCTGAAC-3´ | 20 |
| TraesCS6B01G276300-R | 5´-CTGCATCGACTAGGGCTATG-3´ | 20 |
| TraesCS4A01G143600-F | 5´-AGAGGTGCCATTGACATCGA-3´ | 20 |
| TraesCS4A01G143600-R | 5´-CTTGACAGCATATTCGCCCC-3´ | 20 |
| TraesCS5A01G139700-F | 5´-ACGGCGAGCAAATGCGGAG-3´ | 19 |
| TraesCS5A01G139700-R | 5´-GAGAATTGTACATGGGCAAC-3´ | 20 |
| TraesCS6D01G020700-F | 5´-GTGGGCTTCAACTGGGGCG-3´ | 19 |
| TraesCS6D01G020700-R | 5´-AGCACACGTTGAGGGCCTG-3´ | 19 |
| TraesCS2D01G566900-F | 5´-GCAGGAGCGCGCCGACCTCA-3´ | 20 |
| TraesCS2D01G566900-R | 5´-GTACTTGAGATGATGGAAGA-3´ | 20 |
| Actin-F | 5´-GGATACACGCTTCCTCATGC-3´ | 20 |
| Actin-R | 5´-CTGACAATTTCCCGCTCAGC-3´ | 20 |

F, forward primer; R, reverse primer; BP, base pairs.
