# Supplementary material for: Comparative Transcriptome Analysis of Male Sterile Anthers Induced by High Temperature in Wheat (Triticum aestivum L.)
Source: Front Plant Sci. 2021 Oct 25;12:727966. doi: 10.3389/fpls.2021.727966 (PMC8573241; doi:10.3389/fpls.2021.727966)
Supplement: Supplementary file 2 [file Table_2.docx]

Table S2 Output statistics of sequencing data.

| Sample | Sample name in this article | Clean reads | Clean bases | GC Content | Q20 (%) | Q30 (%) |
| --- | --- | --- | --- | --- | --- | --- |
| Normal anthers | N1 | 28,023,219 | 8,340,017,244 | 55.62% | 96.28 | 91.05 |
| Normal anthers | N 2 | 27,578,382 | 8,226,228,756 | 54.70% | 95.82 | 90.17 |
| Normal anthers | N 3 | 32,679,247 | 9,735,788,800 | 55.42% | 96.15 | 90.82 |
| HT-ms anthers | HT1 | 35,029,597 | 10,441,935,918 | 54.27% | 95.9 | 90.30 |
| HT-ms anthers | HT2 | 30,245,070 | 9,009,651,974 | 54.68% | 95.98 | 90.47 |
| HT-ms anthers | HT3 | 30,153,063 | 8,986,012,058 | 54.19% | 96.05 | 90.61 |

Notes: N and HT stand for the anther samples of normal and high temperature, respectively. 1, 2, and 3 represent the three replicates per sample. GC Content (%)

represents the percentage of Guanine and Cytosine in clean reads. Q30 (%) represents the percentage of nucleotides with quality value greater than or equal to 30.
