# Supplementary material for: Comparative Transcriptome Analysis of Male Sterile Anthers Induced by High Temperature in Wheat (Triticum aestivum L.)
Source: Front Plant Sci. 2021 Oct 25;12:727966. doi: 10.3389/fpls.2021.727966 (PMC8573241; doi:10.3389/fpls.2021.727966)
Supplement: Supplementary file 3 [file Table_3.docx]

Table S3 Summary of the sequence data from Illumina sequencing.

| Sample | Sample name in this article | Replicate | Total reads | Mapped reads | Mapped unique reads | Multiple Map Reads | Reads Map to '+' | Reads Map to '-' |
| --- | --- | --- | --- | --- | --- | --- | --- | --- |
| Normal anthers | N1 | 1 | 56,046,438 | 51,529,162 (91.94%) | 48,425,147 (86.40%) | 3,104,015 (5.54%) | 25,193,439 (44.95%) | 25,404,872 (45.33%) |
| Normal anthers | N2 | 2 | 55,156,764 | 50,303,110 (91.20%) | 47,749,053 (86.57%) | 2,554,057 (4.63%) | 24,698,351 (44.78%) | 24,858,407 (45.07%) |
| Normal anthers | N3 | 3 | 65,358,494 | 59,973,478 (91.76%) | 56,461,804 (86.39%) | 3,511,674 (5.37%) | 29,321,763 (44.86%) | 29,573,367 (45.25%) |
| HT-ms anthers | HT1 | 1 | 70,059,194 | 64,040,780 (91.41%) | 60,498,260 (86.35%) | 3,542,520 (5.06%) | 31,385,605 (44.80%) | 31,614,520 (45.13%) |
| HT-ms anthers | HT2 | 2 | 60,490,140 | 54,996,868 (90.92%) | 51,967,293 (85.91%) | 3,029,575 (5.01%) | 26,941,569 (44.54%) | 27,154,012 (44.89%) |
| HT-ms anthers | HT3 | 3 | 60,306,126 | 54,753,741 (90.79%) | 51,713,265 (85.75%) | 3,040,476 (5.04%) | 26,822,841 (44.48%) | 27,027,757 (44.82%) |

Notes: N and HT stand for the anther samples of normal and high temperature, respectively. 1, 2, and 3 represent the three replicates per sample.
