# Supplementary material for: Comparative Transcriptome Analysis of Male Sterile Anthers Induced by High Temperature in Wheat (Triticum aestivum L.)
Source: Front Plant Sci. 2021 Oct 25;12:727966. doi: 10.3389/fpls.2021.727966 (PMC8573241; doi:10.3389/fpls.2021.727966)
Supplement: Supplementary file 4 [file Table_4.docx]

| GO ID^a^ | Term^b^ | Annotated^c^ | Significant^d^ | Expected^e^ | KS^f^ |
| --- | --- | --- | --- | --- | --- |
| GO:0016023 | cytoplasmic membrane-bounded vesicle | 15118 | 888 | 943.21 | < 1e-30 |
| GO:0000786 | nucleosome | 648 | 185 | 40.43 | < 1e-30 |
| GO:0005886 | plasma membrane | 7700 | 478 | 480.4 | 1.90E-13 |
| GO:0043231 | intracellular membrane-bounded organelle | 49205 | 2889 | 3069.88 | 2.90E-10 |
| GO:0005618 | cell wall | 2580 | 210 | 160.97 | 3.20E-09 |
| GO:0009507 | chloroplast | 8197 | 337 | 511.41 | 3.30E-08 |
| GO:0031969 | chloroplast membrane | 408 | 17 | 25.45 | 1.10E-07 |
| GO:0071944 | cell periphery | 9603 | 660 | 599.13 | 4.60E-07 |
| GO:0009536 | plastid | 16410 | 894 | 1023.81 | 9.30E-07 |
| GO:0044444 | cytoplasmic part | 45248 | 2624 | 2823.01 | 1.50E-06 |

Table S4 The enrichment results for the cellular component DEGs by topGO.

Note: go Note: ^a^GO term ID; ^b^GO function; ^c^all genes annotated the function; ^d^DEGs annotated the function; ^e^Expected value of the DEGs annotated the function; ^f^Statistical significance of enrichment nodes, the smaller the KS value, the more significant enrichment.
