# Supplementary material for: Comparative Transcriptome Analysis of Male Sterile Anthers Induced by High Temperature in Wheat (Triticum aestivum L.)
Source: Front Plant Sci. 2021 Oct 25;12:727966. doi: 10.3389/fpls.2021.727966 (PMC8573241; doi:10.3389/fpls.2021.727966)
Supplement: Supplementary file 5 [file Table_5.docx]

Table S5 The enrichment results for the biological process DEGs by topGO.

| GO ID^a^ | Term^b^ | Annotated^c^ | Significant^d^ | Expected^e^ | KS^f^ |
| --- | --- | --- | --- | --- | --- |
| GO:0006334 | nucleosome assembly | 642 | 182 | 41.38 | < 1e-30 |
| GO:0009651 | response to salt stress | 1567 | 80 | 100.99 | 2.80E-10 |
| GO:0046686 | response to cadmium ion | 1419 | 62 | 91.46 | 1.10E-09 |
| GO:0006270 | DNA replication initiation | 166 | 38 | 10.7 | 1.30E-09 |
| GO:0015798 | myo-inositol transport | 28 | 1 | 1.8 | 2.40E-09 |
| GO:0043086 | negative regulation of catalytic activity | 681 | 69 | 43.89 | 2.70E-09 |
| GO:0055114 | oxidation-reduction process | 8114 | 411 | 522.95 | 6.70E-09 |
| GO:0009860 | pollen tube growth | 348 | 55 | 22.43 | 7.30E-09 |
| GO:0009715 | chalcone biosynthetic process | 27 | 1 | 1.74 | 1.20E-08 |
| GO:0008283 | cell proliferation | 547 | 98 | 35.25 | 1.90E-08 |

Note: go Note: ^a^GO term ID; ^b^GO function; ^c^all genes annotated the function; ^d^DEGs annotated the function; ^e^Expected value of the DEGs annotated the function; ^f^Statistical significance of enrichment nodes, the smaller the KS value, the more significant enrichment.
