# Supplementary material for: Comparative Transcriptome Analysis of Male Sterile Anthers Induced by High Temperature in Wheat (Triticum aestivum L.)
Source: Front Plant Sci. 2021 Oct 25;12:727966. doi: 10.3389/fpls.2021.727966 (PMC8573241; doi:10.3389/fpls.2021.727966)
Supplement: Supplementary file 6 [file Table_6.docx]

Table S6 The enrichment results for the molecular function DEGs by topGO.

| GO ID^a^ | Term^b^ | Annotated^c^ | Significant^d^ | Expected^e^ | KS^f^ |
| --- | --- | --- | --- | --- | --- |
| GO:0046982 | protein heterodimerization activity | 773 | 179 | 48.96 | 5.70E-26 |
| GO:0004601 | peroxidase activity | 863 | 51 | 54.66 | 2.50E-13 |
| GO:0020037 | heme binding | 2362 | 126 | 149.59 | 4.10E-13 |
| GO:0045330 | aspartyl esterase activity | 101 | 25 | 6.4 | 5.40E-11 |
| GO:0004713 | protein tyrosine kinase activity | 707 | 57 | 44.78 | 1.10E-10 |
| GO:0030599 | pectinesterase activity | 227 | 35 | 14.38 | 1.10E-08 |
| GO:0016210 | naringenin-chalcone synthase activity | 32 | 1 | 2.03 | 1.30E-08 |
| GO:0015020 | glucuronosyltransferase activity | 65 | 0 | 4.12 | 3.80E-08 |
| GO:0042973 | glucan endo-1,3-beta-D-glucosidase activity | 96 | 14 | 6.08 | 4.80E-08 |
| GO:0009055 | electron carrier activity | 2495 | 129 | 158.01 | 5.50E-08 |

Note: go Note: ^a^GO term ID; ^b^GO function; ^c^all genes annotated the function; ^d^DEGs annotated the function; ^e^Expected value of the DEGs annotated the function; ^f^Statistical significance of enrichment nodes, the smaller the KS value, the more significant enrichment.
