# Supplementary figures and images for: Comparative Transcriptome Analysis of Male Sterile Anthers Induced by High Temperature in Wheat (Triticum aestivum L.)
Source: Front Plant Sci. 2021 Oct 25;12:727966. doi: 10.3389/fpls.2021.727966 (PMC8573241; doi:10.3389/fpls.2021.727966)

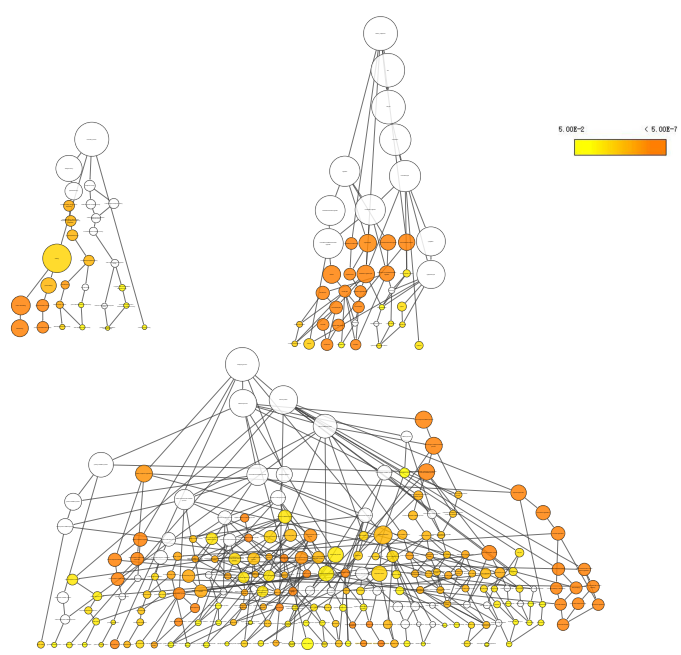

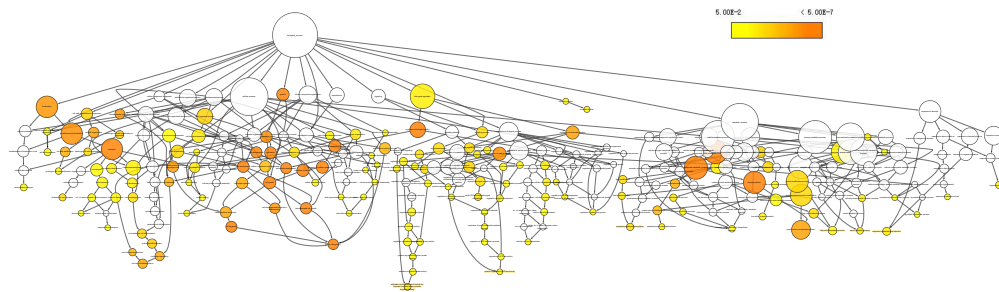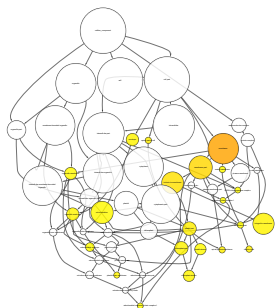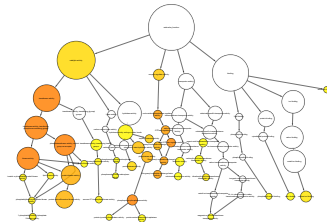

Supplement: Supplementary file 9 [file Data_Sheet_3.pdf]

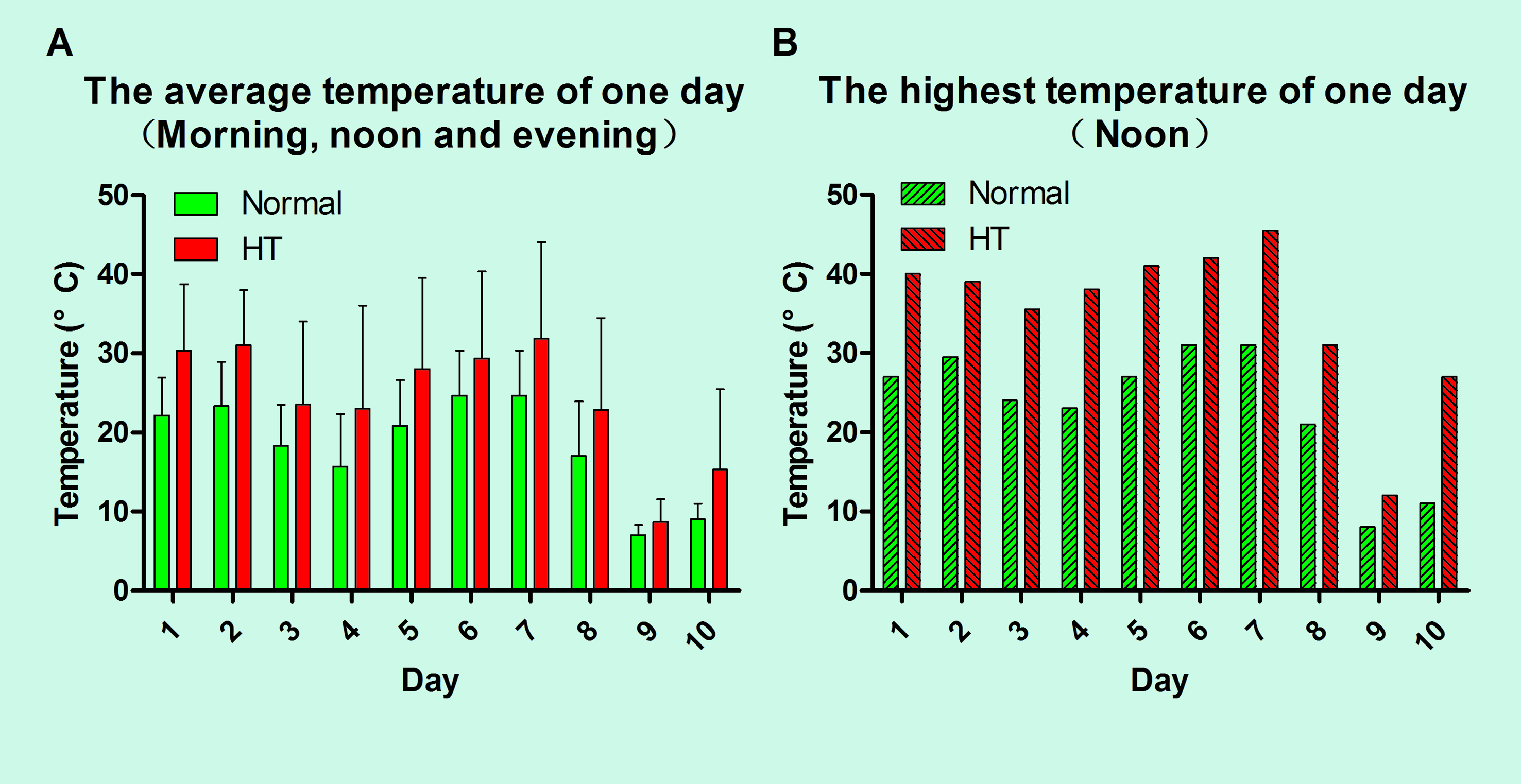

Supplement: Supplementary file 12 [file Image_1.TIF]

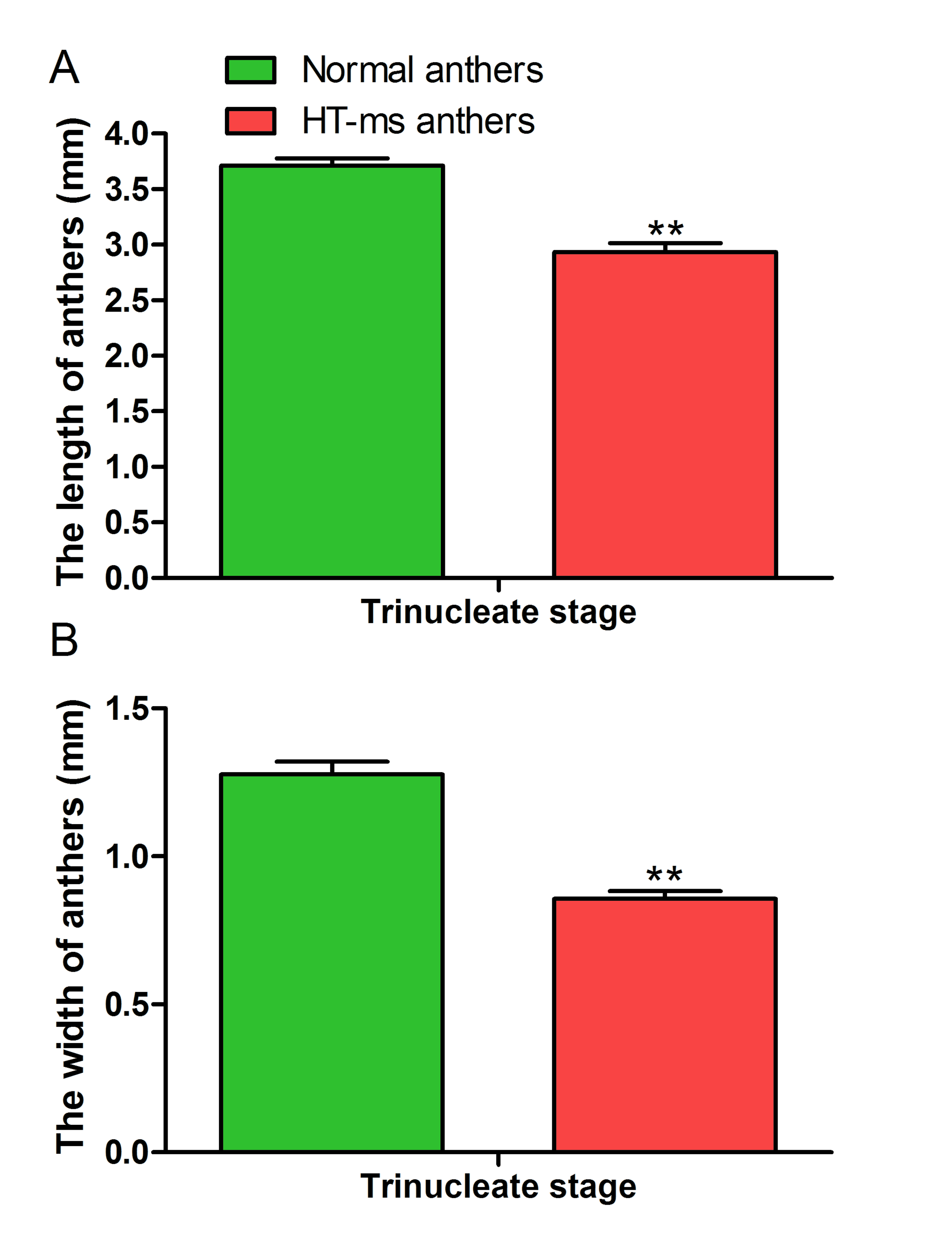

Supplement: Supplementary file 13 [file Image_2.TIF]

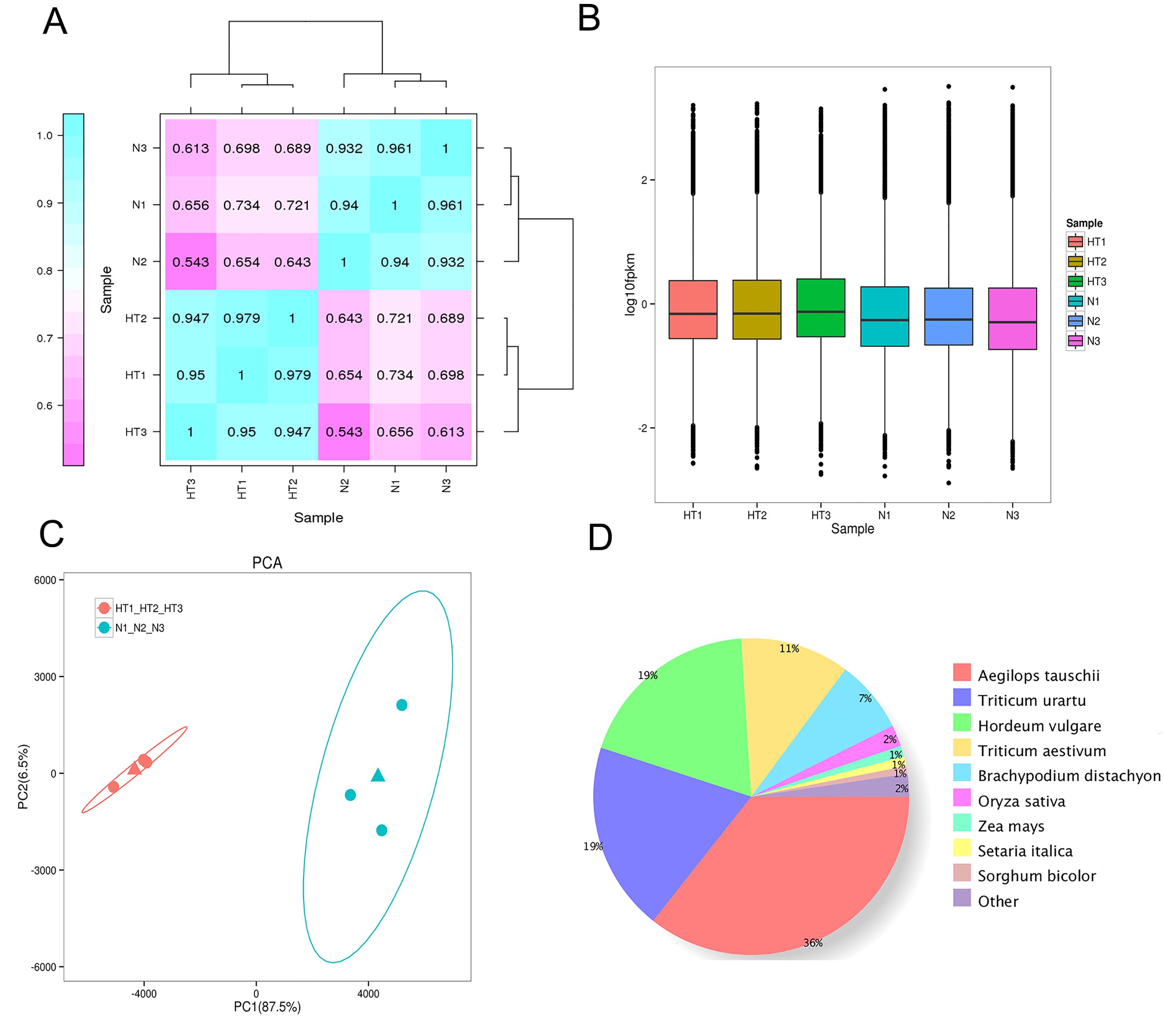

Supplement: Supplementary file 14 [file Image_3.TIF]

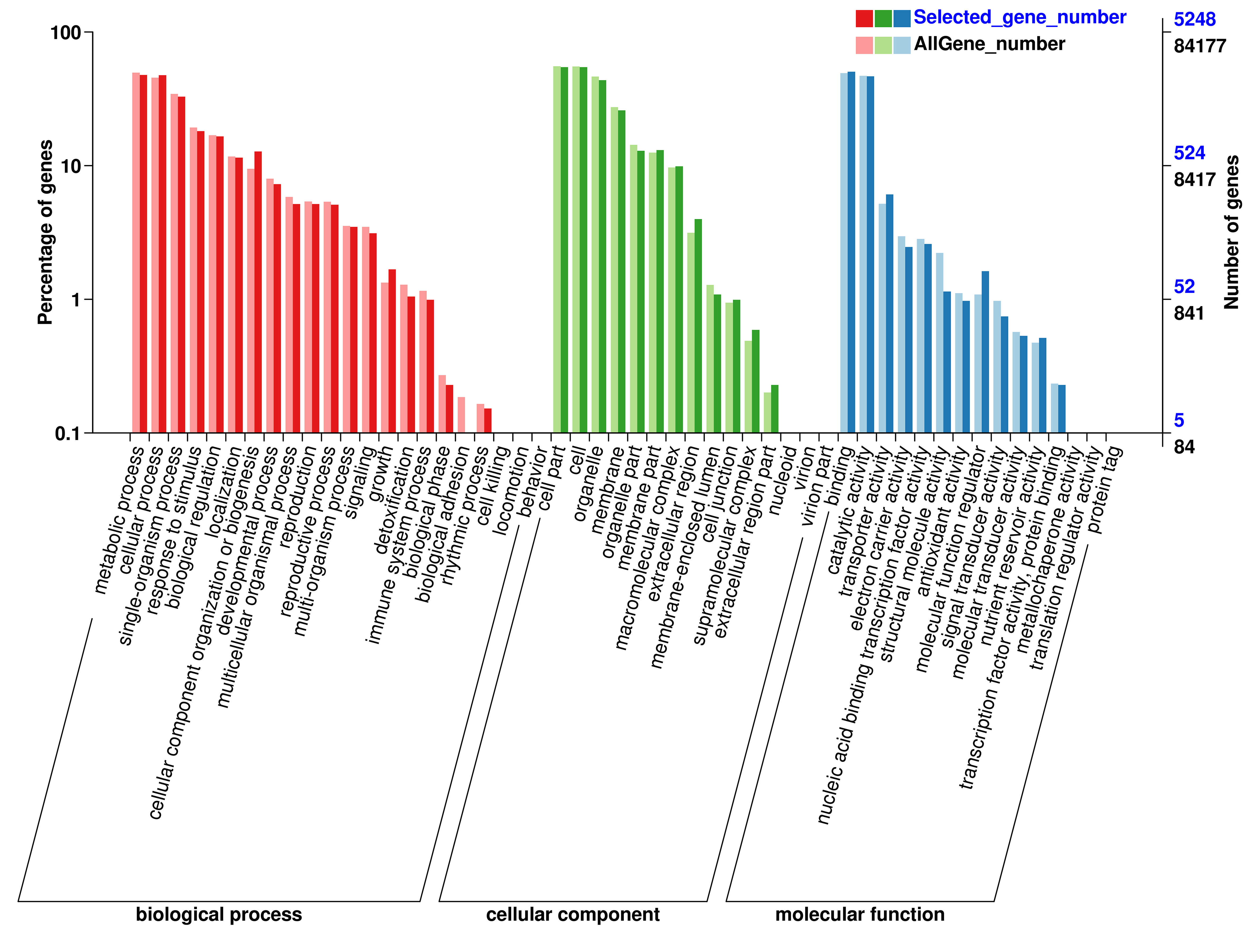

Supplement: Supplementary file 15 [file Image_4.TIF]

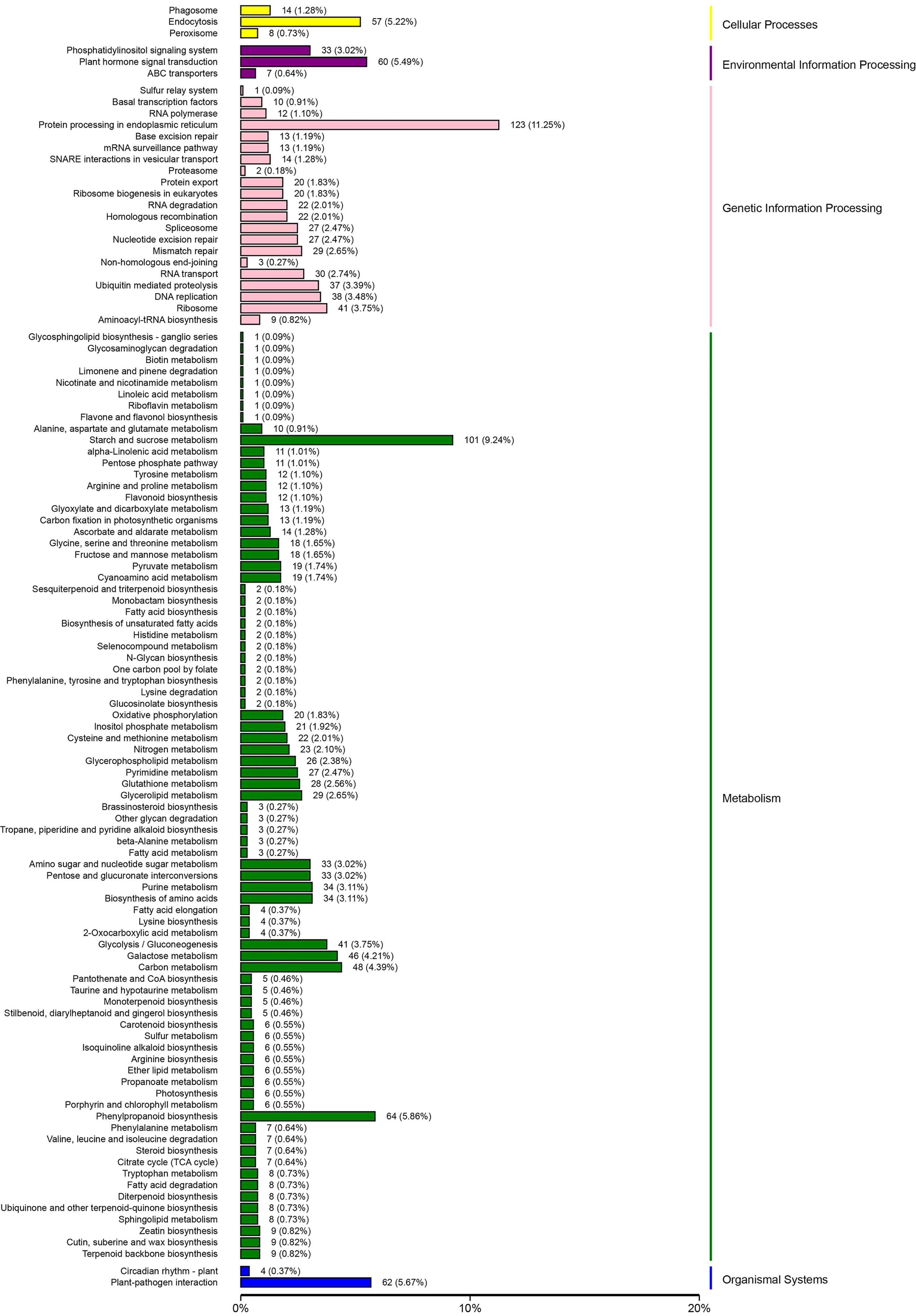

Supplement: Supplementary file 16 [file Image_5.TIF]

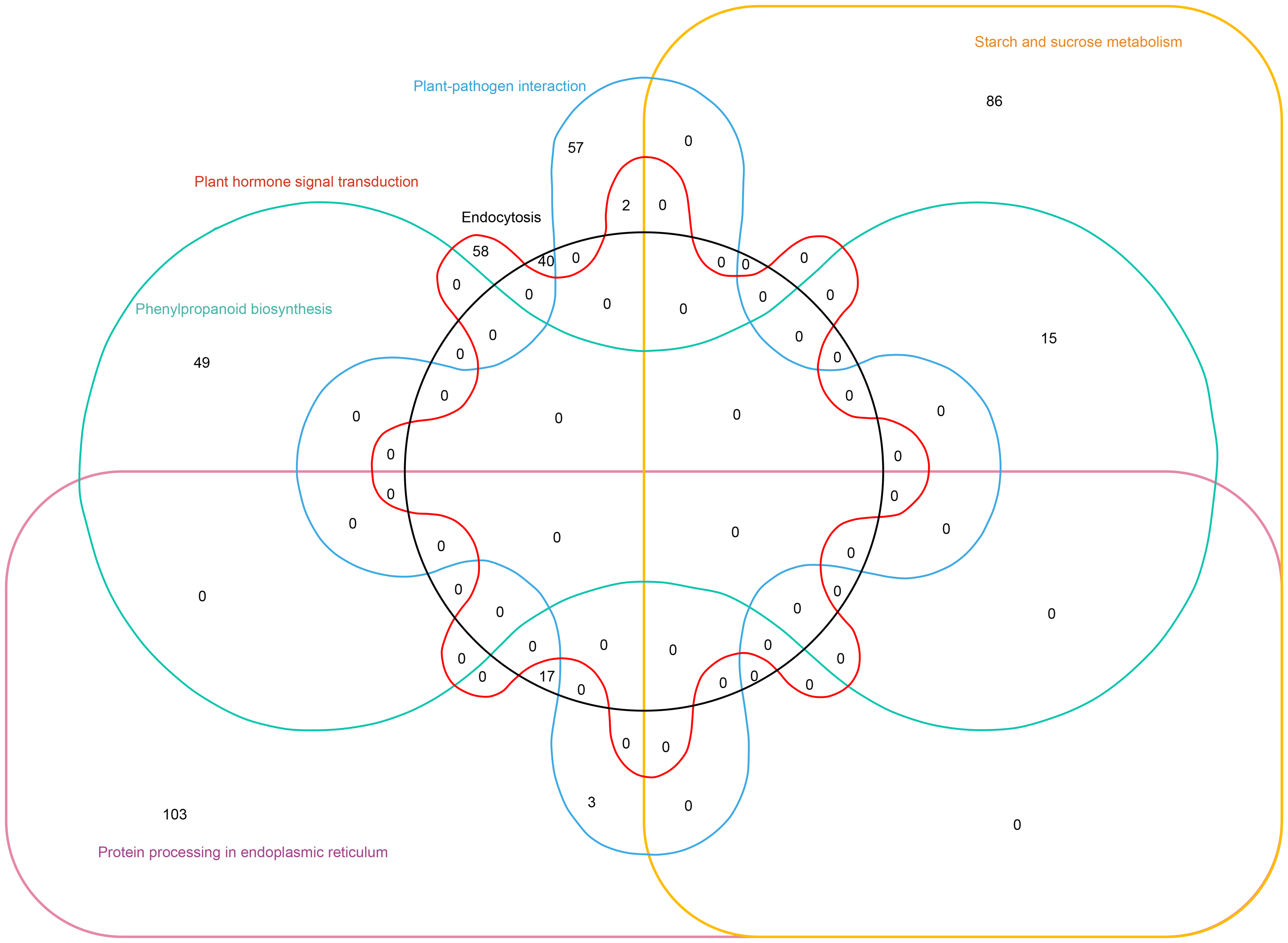

Supplement: Supplementary file 17 [file Image_6.TIF]

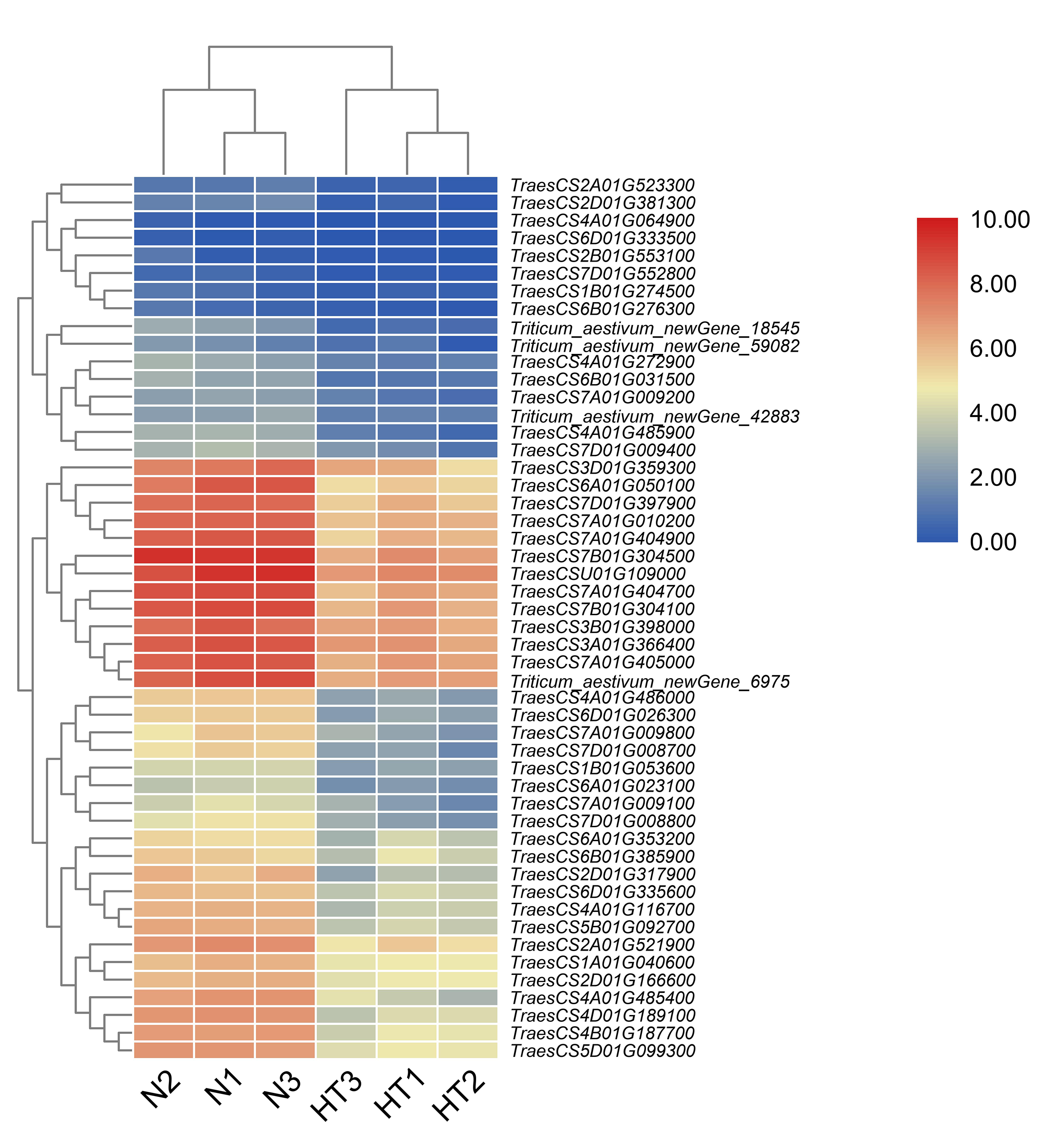

Supplement: Supplementary file 18 [file Image_7.TIF]

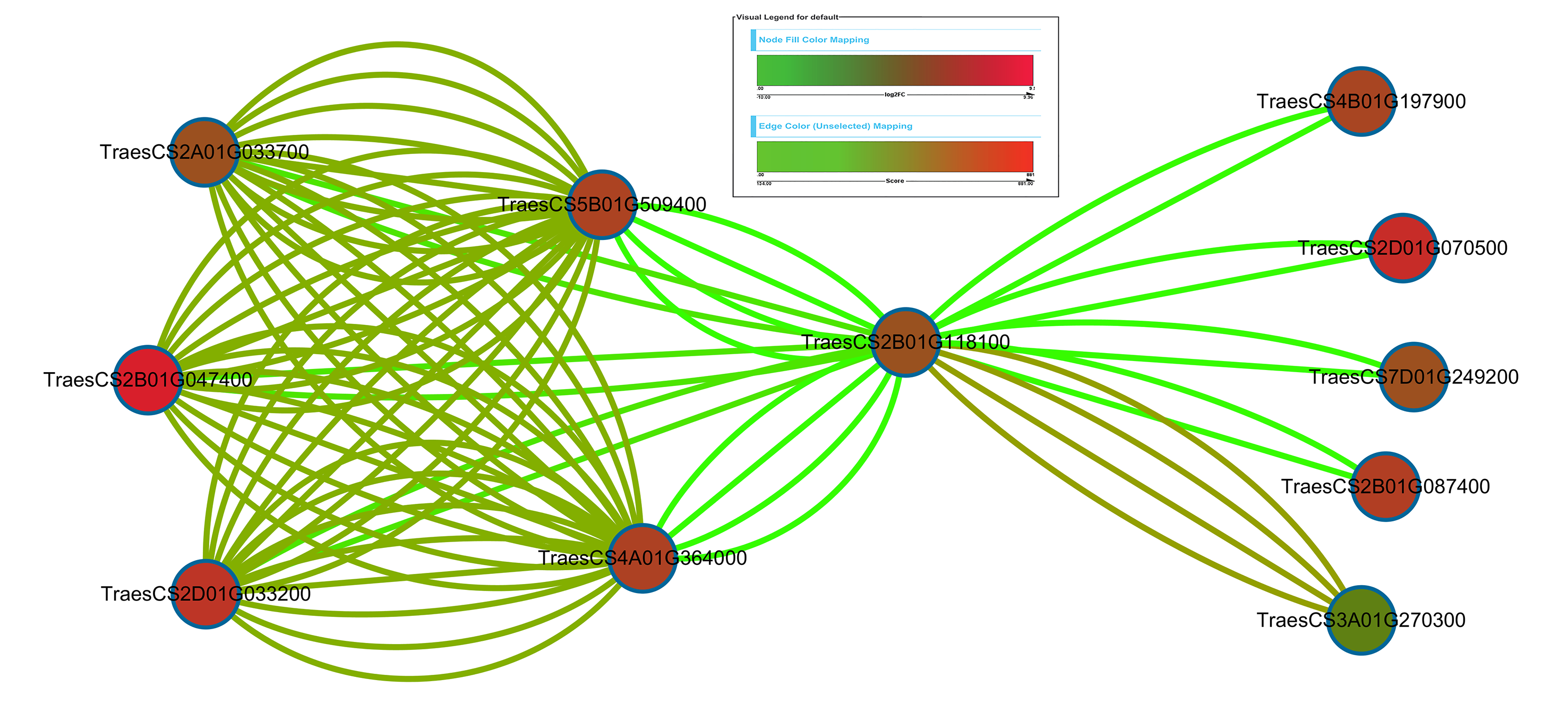

Supplement: Supplementary file 19 [file Image_8.TIF]

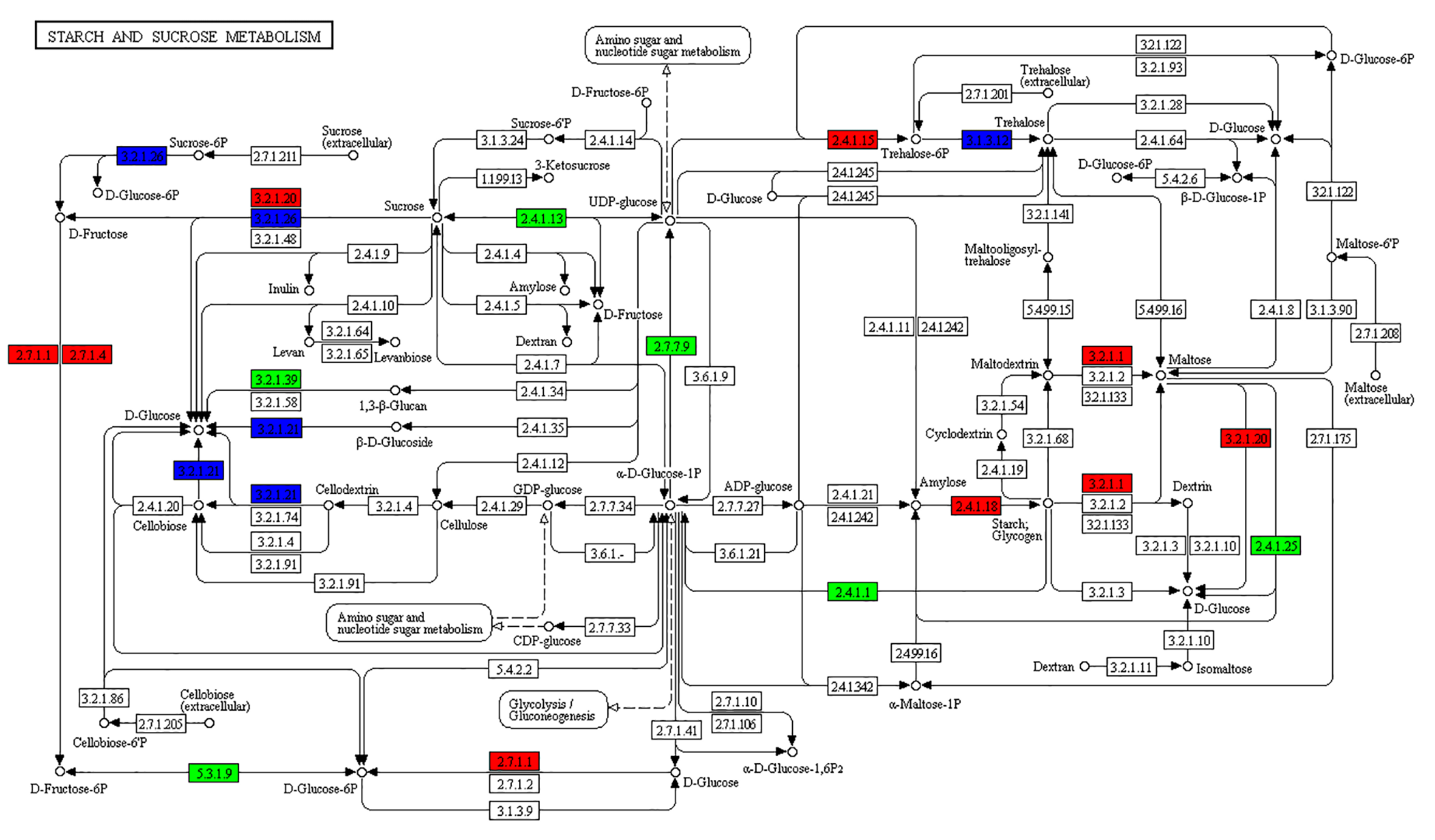

Supplement: Supplementary file 20 [file Image_9.TIF]

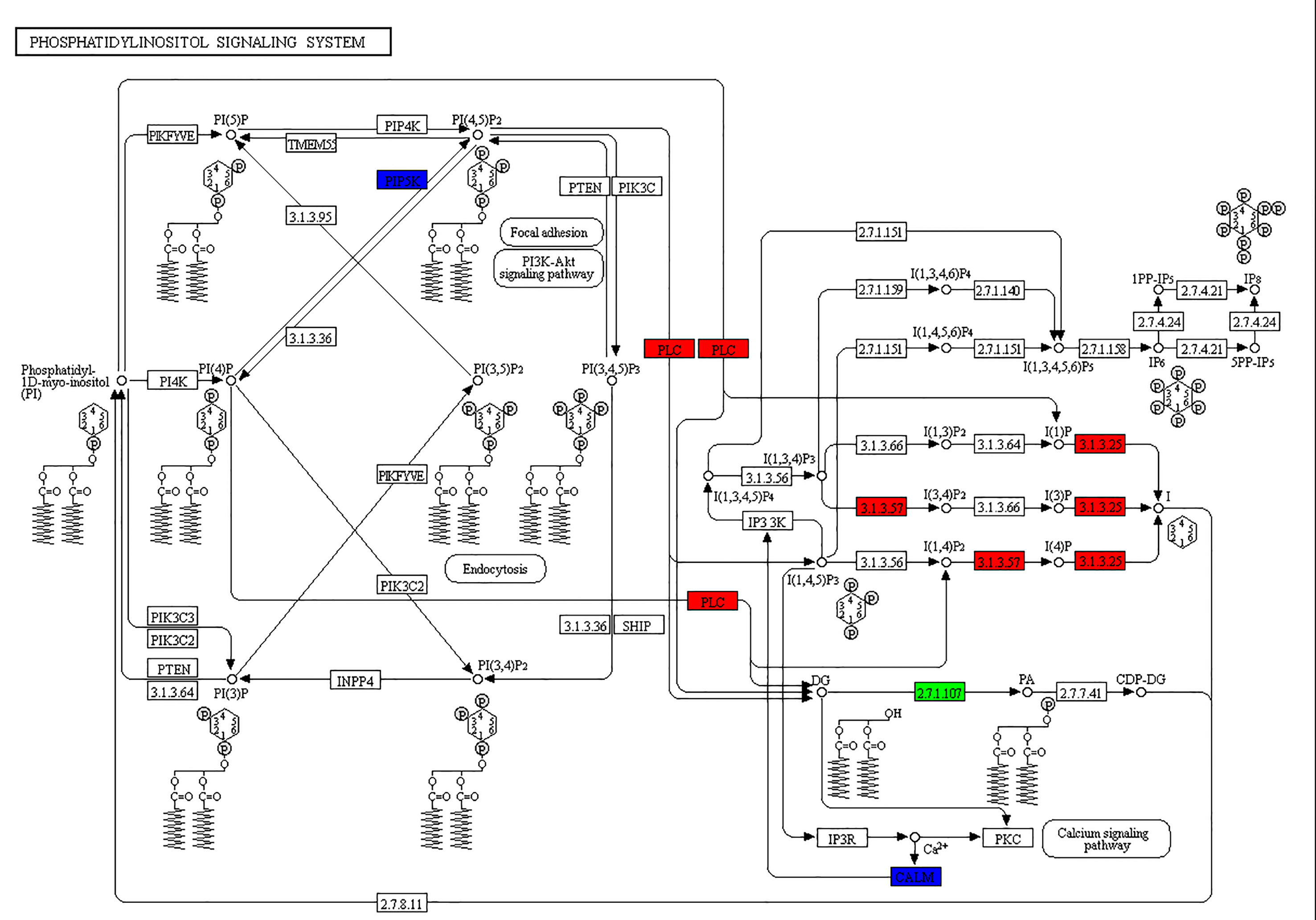

Supplement: Supplementary file 21 [file Image_10.TIF]
