# Supplementary material for: Comparative Transcriptome Analysis of Male Sterile Anthers Induced by High Temperature in Wheat (Triticum aestivum L.)
Source: Front Plant Sci. 2021 Oct 25;12:727966. doi: 10.3389/fpls.2021.727966 (PMC8573241; doi:10.3389/fpls.2021.727966)

Peroxidase activity and response to oxidative stress

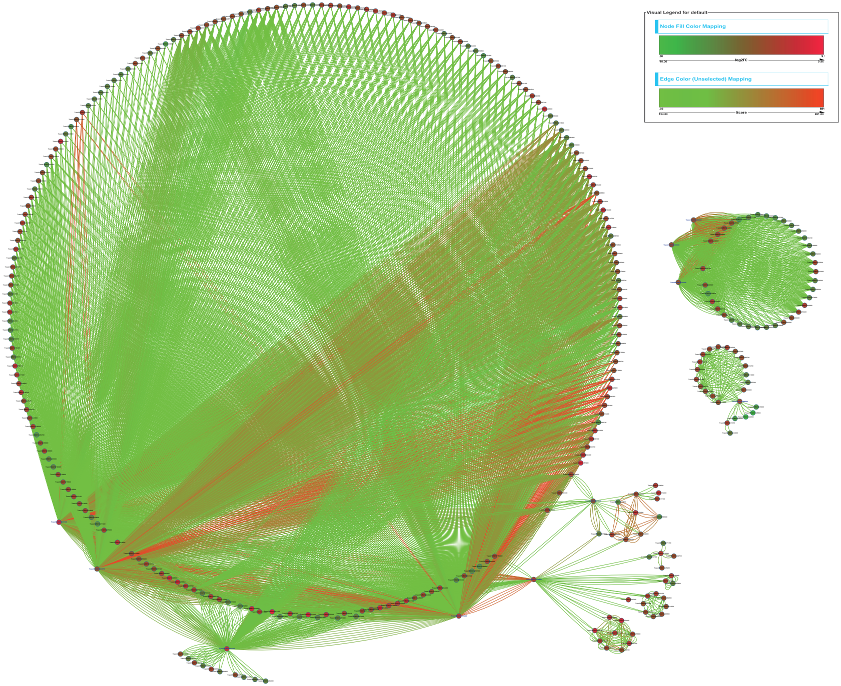

Starch and sucrose metabolism

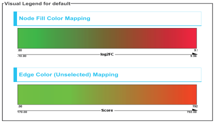

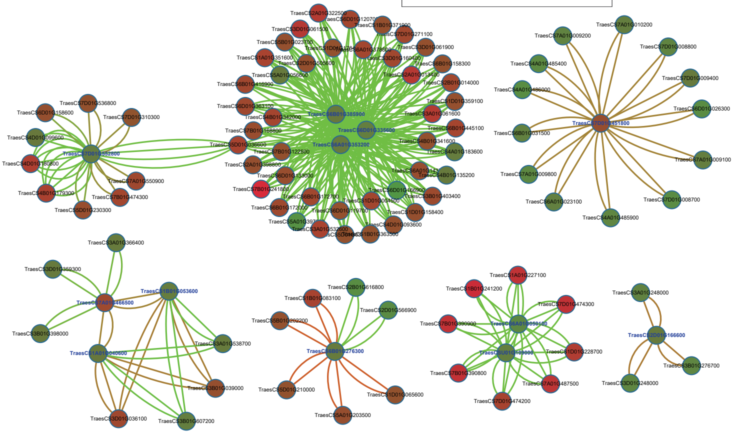

Phosphatidylinositol signaling system

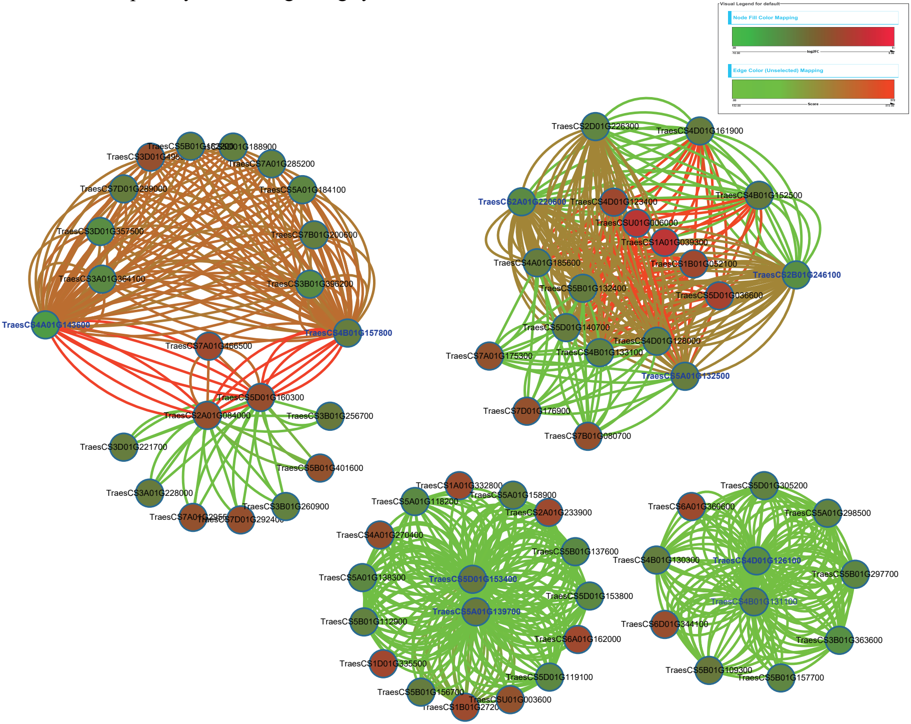

Heme binding

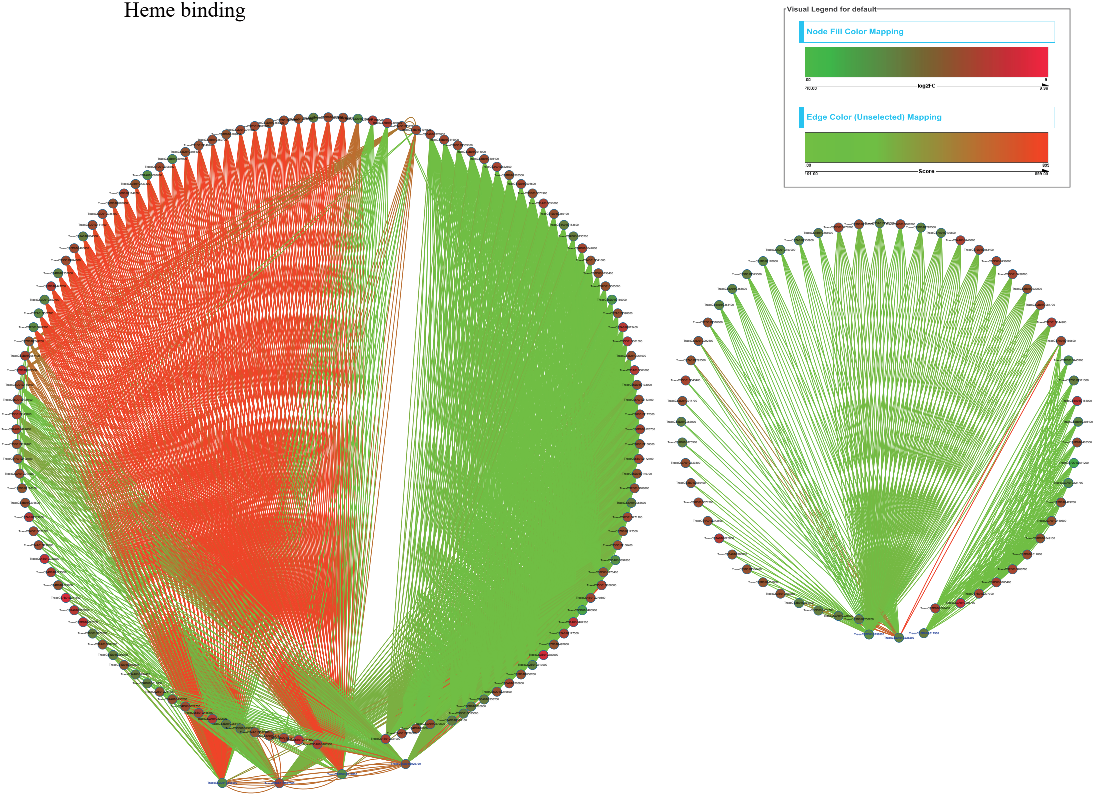

Supplement: Supplementary file 11 [file Data_Sheet_5.pdf]
